# MicroRNAs in extracellular vesicles released from epicardial adipose tissue promote arrhythmogenic conduction slowing

**DOI:** 10.1016/j.hroo.2023.10.007

**Published:** 2023-11-02

**Authors:** Auriane C. Ernault, Rosan de Winter, Benedetta Fabrizi, Jillian W.P. Bracht, Chi Hau, Shirley C.M. van Amersfoorth, Eva R. Meulendijks, Anke J. Tijsen, Lucía Cócera Ortega, Ingeborg van der Made, Aleksandra Gasecka, Antoine H. Driessen, Rienk Nieuwland, Bastiaan J. Boukens, Edwin van der Pol, Joris R. de Groot, Ruben Coronel

**Affiliations:** ∗ Department of Clinical and Experimental Cardiology, Amsterdam UMC, location University of Amsterdam, Amsterdam, the Netherlands; † Heart Failure and Arrhythmias, Amsterdam Cardiovascular Sciences, Amsterdam, the Netherlands; ‡ Laboratory of Experimental Clinical Chemistry, Amsterdam UMC, location AMC, Amsterdam, the Netherlands; § Vesicle Observation Center, Amsterdam UMC, location AMC, Amsterdam, the Netherlands; || Biomedical Engineering and Physics, Amsterdam UMC, location AMC, Amsterdam, the Netherlands; ¶ Department of Cardiology, Medical University of Warsaw, Warsaw, Poland; ∗∗ Department of Medical Biology, Amsterdam UMC, location University of Amsterdam, Amsterdam, the Netherlands

**Keywords:** Obesity, Atrial fibrillation, Epicardial adipose tissue, Arrhythmias, Adiposity, miRNA

## Abstract

**Background:**

Patients with excess epicardial adipose tissue (EAT) are at increased risk of developing cardiac arrhythmias. EAT promotes arrhythmias by depolarizing the resting membrane of cardiomyocytes, which slows down conduction and facilitates re-entrant arrhythmias. We hypothesized that EAT slows conduction by secreting extracellular vesicles (EVs) and their microRNA (miRNA) cargo.

**Objective:**

We aimed to determine the role of EAT-derived EVs and their miRNA cargo in conduction slowing.

**Methods:**

EAT and subcutaneous adipose tissue (SAT) were collected from patients with atrial fibrillation. Adipose tissue explants were incubated in culture medium and secretome was collected. The numbers of EVs in the EAT and SAT secretome were measured by calibrated flow cytometry. EVs in the EAT secretome were isolated by size exclusion chromatography and miRNAs were sequenced. Pathway analysis was performed to predict candidates involved in cardiac electrophysiology. The candidates were validated in the EAT and SAT by quantitative real-time polymerase chain reaction. Finally, miRNA candidates were overexpressed in neonatal rat ventricular myocytes.

**Results:**

The EV concentration was higher in the EAT secretome than in the SAT and control secretomes. miRNA sequencing of EAT-derived EVs detected a total of 824 miRNAs. Pathway analysis led to the identification of 7 miRNAs potentially involved in regulation of cardiac resting membrane potential. Validation of those miRNA candidates showed that they were all expressed in EAT, and that miR-1-3p and miR-133a-3p were upregulated in EAT in comparison with SAT. Overexpression of miR-1-3p and miR-133a-3p in neonatal rat ventricular myocytes led to conduction slowing and reduced *Kcnj2* and *Kcnj12* expression.

**Conclusion:**

miR-1-3p and miR-133a-3p are potential mediators of EAT arrhythmogenicity.

**Key Findings**

▪ Extracellular vesicle concentration is higher in epicardial than in subcutaneous adipose tissue secretome.
▪ Epicardial adipose tissue secretome is rich in extracellular vesicles containing microRNAs predicted to regulate resting membrane potential.
▪ miR-1-3p and miR-133a-3p induce arrhythmogenic features when transfected into neonatal rat ventricular myocytes.

## Introduction

The rates of overweight and obesity have increased globally in the last decade and are expected to keep rising.^1^ It is predicted that 1 in 5 adults will be obese by 2025.^1^ Obesity is an independent risk factor for atrial fibrillation (AF),^2^ which is associated with ischemic stroke, heart failure, cognitive decline, and increased mortality.^3^

In obese individuals, adipose tissue accumulates around internal organs including the heart. The absence of fascia separating epicardial adipose tissue (EAT) from the myocardium allows paracrine exchange between the 2 tissues.^4^ Paracrine communication is illustrated by the arrhythmogenic effect of EAT secretome on cardiac conduction and repolarization,^5^ which are both parameters involved in re-entry and subsequent AF. Identification of the components secreted by EAT that are responsible for this arrhythmogenic electrical remodeling can provide novel antiarrhythmic therapeutic targets.

The secretome of EAT contains extracellular vesicles (EVs), a family of cell-secreted lipid vesicles including microvesicles, exosomes and apoptotic bodies known to mediate intercellular communication.^6^ EAT-derived EVs shorten the action potential duration at 80% repolarization and induce sustained rotors in human induced pluripotent stem cell–based cardiac cell sheets.^7^

EVs carry regulatory molecules such as microRNAs (miRNAs) that have been implicated in various physiological and pathological processes.^8^ miRNAs are short noncoding RNAs that mediate gene expression posttranscriptionally. By binding to the 3′ untranslated region (UTR) of messenger RNA, miRNAs can inhibit translation or cause messenger RNA degradation.^9^ miRNAs transported by EVs have been found to directly regulate gene expression in targeted cells.^10^ However, the effect of miRNAs in EAT-derived EVs on cardiomyocytes is not sufficiently understood.

We previously showed that secretome of EAT but not of subcutaneous adipose tissue (SAT) from AF patients reduces expression of *Kcnj2*. *Kcnj2* is a gene encoding Kir2.1, which is a subunit of the K^+^ channel responsible for the inward rectifier K^+^ current during cardiac repolarization. The reduced expression of *Kcnj2* induced by EAT secretome led to depolarization of the resting membrane and arrhythmogenic conduction slowing in neonatal rat ventricular myocytes (NRVMs).^5^

Here, we hypothesized that EAT secretes EVs containing miRNAs that induce arrhythmogenic remodeling of cardiomyocytes. The aim of this study was to quantify EAT- and SAT-derived EVs in the secretome, characterize the miRNA cargo of EAT-derived EVs, and assess the effect of these miRNAs on conduction.

## Methods

An extended version of the method is available in the Supplemental Material.

### Statistics

Statistical analyses were performed with GraphPad Prism (V8, GraphPad Software, San Diego, CA). Data are given as mean ± SD unless indicated otherwise. Number of observations and repeated experiments are given in the figure legends. Data were tested for normality using a Shapiro-Wilk normality test. If normal distribution was verified, the 2-tailed Student’s *t* test was performed. If the data were not normally distributed, statistical significance was assessed using the 2-tailed Mann-Whitney *U* test or 1-way analysis of variance and post hoc test. *P* ≤ .05 was considered significant.

## Results

### EAT releases more EVs than SAT

**Figure 1:**
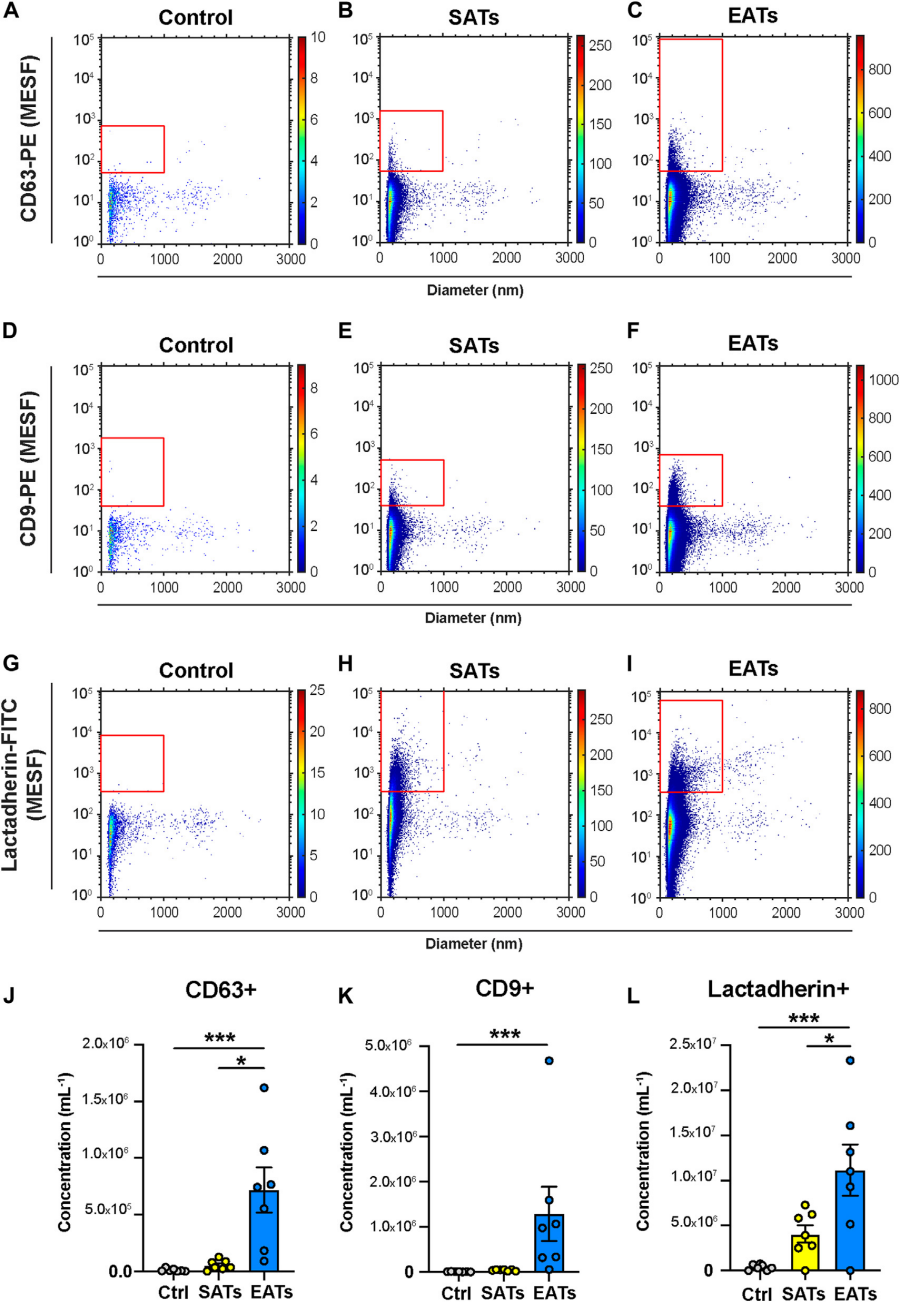
Extracellular vesicle (EVs) concentrations measured by flow cytometry differ between epicardial adipose tissue (EAT) and subcutaneous adipose tissue (SAT) secretomes. **A–I:** Fluorescence intensity vs diameter of extracellular vesicles labeled with anti-CD63 **(A–C)** and anti-CD9 **(D–F)** antibodies conjugated to phycoerythrin (PE), and lactadherin **(G–I)** antibody conjugated to FITC, in control culture medium, SATs and EATs from 1 patient. Note that in this example the EATs were diluted 2-fold in phosphate-buffered saline before antibody staining (cf. Methods). **J–L:** Concentrations of EVs labeled with anti-CD63-PE **(J)**, anti-CD9-PE **(K)**, and lactadherin-FITC **(L)** in control culture medium and SATs and EATs obtained from n = 7 patients. Concentrations of EVs show the number of particles per mL of secretome (1) exceeding the side scattering threshold, (2) having a diameter <1000 nm, and (3) exceeding the fluorescent gate corresponding to the used labels. Data are mean ± SEM. One-way analysis of variance and Tukey post hoc test for lactadherin, nonparametric Kruskal-Wallis test and Dunn's post hoc test for CD63 and CD9. Method details are found in the Supplemental Material (MIFlowCyt-EV); Supplemental Tables 5, 6, and 7; and Supplemental Figures 6 and 7. ∗*P* < .05, ∗∗∗*P* < .001. Ctrl, control; MESF = molecules of equivalent soluble fluorochrome.

We first quantified the EV concentration in EAT and SAT secretome samples obtained from 7 patients by flow cytometry, using antibodies against EV markers CD9 and CD63, and lactadherin. Figure 1 shows representative examples of EV measurement obtained from 1 patient for CD63-PE (Figures 1A–1C), CD9-PE (Figures 1D–1F), and lactadherin-FITC (Figures 1G–1I) in control culture medium, and in SAT and EAT secretome samples.

Overall, the concentration of CD63+ EVs was 1.01 × 10^4^ ± 1.32 × 10^4^ EVs/mL for the control, 5.46 × 10^4^ ± 4.43 × 10^4^ EVs/mL for the SAT secretome, and 7.17 × 10^5^ ± 5.24 × 10^5^ EVs/mL for the EAT secretome (Figure 1J). The concentration of CD63+ EVs was significantly higher in the EAT secretome than the SAT secretome (*P < .*05, n = 7) and control (*P < .*001, n = 7).

The average concentration of CD9+ EVs was 7.20 × 10^3^ ± 4.47 × 10^3^ EVs/mL for the control, 4.15 × 10^4^ ± 1.74 × 10^4^ EVs/mL for the SAT secretome, and 1.29 × 10^6^ ± 1.59 × 10^6^ EVs/mL for the EAT secretome (Figure 1K). The concentration of CD9+ EVs was significantly larger in the EAT secretome than the control secretome (*P < .*001, n = 7) but was not larger than the SAT secretome.

Finally, the average concentration of lactadherin+ EVs was 3.81 × 10^5^ ± 3.15 × 10^5^ EVs/mL for the control secretome, 4.08 × 10^6^ ± 2.54 × 10^6^ EVs/mL for the SAT secretome, and 1.11 × 10^7^ ± 7.52 × 10^6^ EVs/mL for the EAT secretome (Figure 1L). The concentration of lactadherin+ EVs was significantly larger in the EAT secretome than the SAT secretome (*P < .*05, n = 7) and the control secretome (*P < .*001, n = 7).

### EAT secretome–derived EVs contain miRNAs predicted to regulate the resting membrane potential

**Figure 2:**
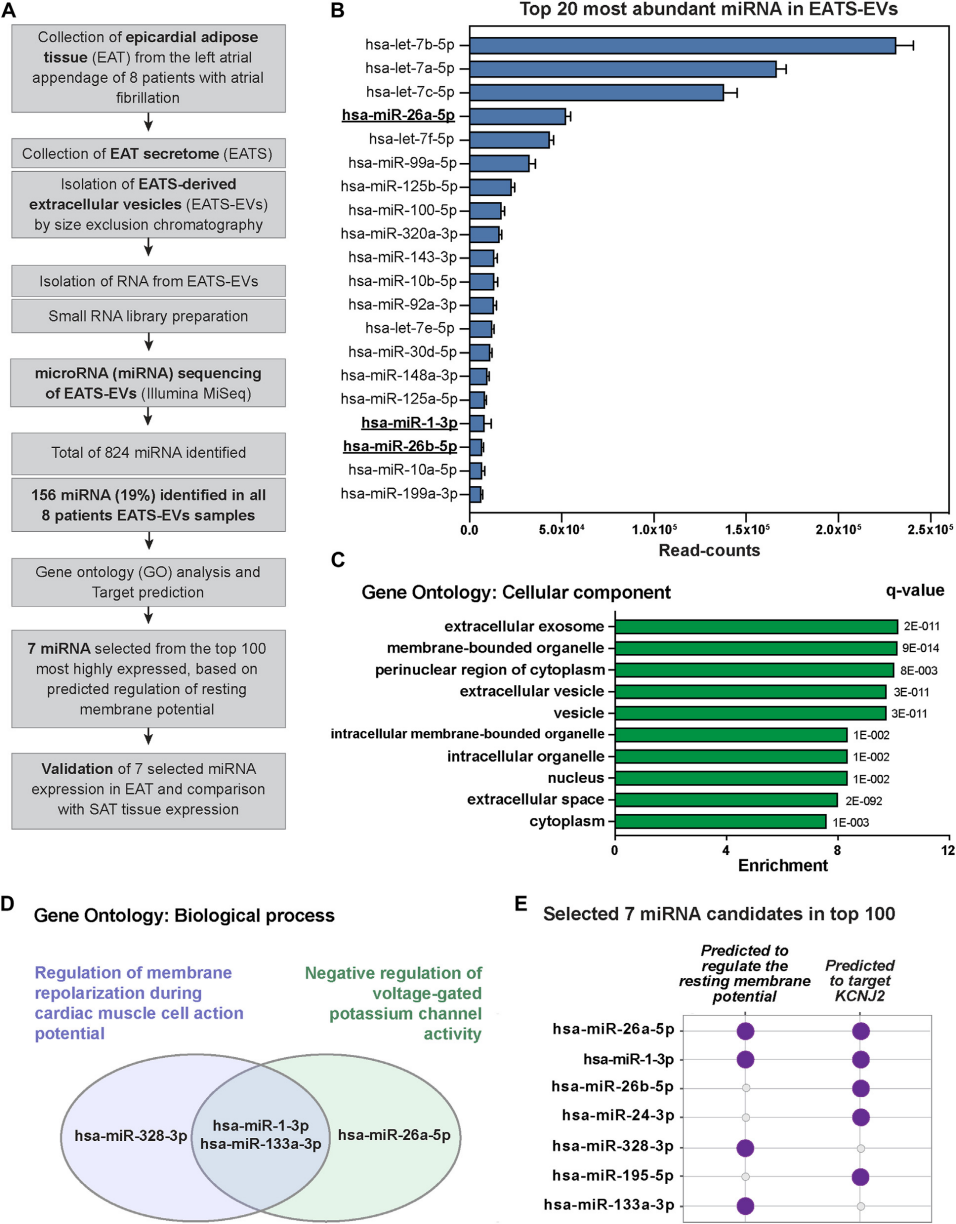
Epicardial adipose tissue secretome–derived extracellular vesicles (EATS-EVs) contain several microRNA (miRNA) predicted to regulate the resting membrane potential. **A:** Flow chart depicting the methodology used to characterize miRNA cargos in EATS-EVs. **B:** Top 20 most abundant miRNAs in EATS-EVs among the 156 miRNAs identified in all 8 EATS-EV samples. miRNA predicted to target *KCNJ2* (cf. panel **E**) are shown in bold and underlined (hsa-miR-26a-5p, hsa-miR-1-3p, and hsa-miR-26b-5p). **C:** Gene Ontology (GO) analysis indicating the top 10 most highly enriched “cellular component” terms and q value. To perform this analysis, the 156 miRNAs identified in all 8 patients were used as input in GeneTrail2. The indicated q value corresponds to the adjusted *P* value for multiple testing using the Benjamin-Yekutieli method. **D:** miRNA hits for the enriched biological process GO: “Regulation of membrane repolarization during cardiac muscle cell action potential” (enrichment = 16.7, q value = 2.87 × 10^-3^) and “Negative regulation of voltage-gated potassium channel activity” (enrichment = 10, q value = 2.06 × 10^-2^). **E:** miRNA selected for validation in EAT and comparison with SAT, based on (1) predicted regulation of resting membrane potential (hits from GO biological process analysis identified in **D**), (2) predicted interaction with *KCNJ2* (TargetScan analysis), and (3) detection level (top 100). SAT = subcutaneous adipose tissue.

To characterize the miRNA cargo of EAT secretome–derived EVs, we isolated EVs from EAT secretome of 8 patients (patients characteristics provided in Supplemental Table 1; secretome sample concentration in Supplemental Figure 1) by size exclusion chromatography and performed miRNA sequencing (workflow in Figure 2A). By miRNA sequencing, we detected a total of 824 miRNAs, of which 156 (19%) miRNAs were commonly expressed in the 8 patients with EAT secretome–derived EVs with at least 5 read counts in all samples (Supplemental Table 2). Figure 2B shows the top 20 miRNAs with the highest read counts in EVs.

To identify the main processes regulated by these 156 miRNA, we performed Gene Ontology (GO) enrichment analysis using GeneTrail2.^11^ In the GO category “cellular component,” we observed a significant enrichment for EV-related terms such as “extracellular exosome,” “membrane-bounded organelle,” “extracellular vesicle,” “vesicle,” and “extracellular space” (Figure 2C).

We previously reported that the EAT secretome from AF patients reduces expression of *Kcnj2*, leading to depolarization of the resting membrane and arrhythmogenic conduction slowing in NRVMs.^5^ Therefore, we aimed to narrow the list of miRNA candidates to electrophysiologically relevant miRNAs, especially those relevant for resting membrane potential and potassium channel regulation. To do so, we used the GO category “Biological process,” which revealed an enrichment for “Regulation of membrane repolarization during cardiac muscle cell action potential” (enrichment = 16.7, q value = 2.87 × 10^–3^) and “Negative regulation of voltage-gated potassium channel activity” (enrichment = 10, q value = 2.06 × 10^–2^), which contained miR-328-3p, miR-1-3p, miR-133a-3p, and miR-26a-5p (Figures 2D and 2E).

To make sure that all miRNAs potentially involved in regulation of the resting membrane potential were identified from our dataset, we also performed target prediction analysis on *KCNJ2* using the top 100 miRNAs with the highest read counts in EVs (TargetScan).^12^ This prediction revealed 5 miRNAs that could target *KCNJ2*: miR-26a-5p, miR-1-3p, miR-26b-5p, miR-24-3p, and miR-195-5p (Figure 2E). Among these 5 miRNAs predicted to target *KCNJ2*, 2 miRNAs (miR-26a-5p and miR-1-3p) were already identified in the GO enriched terms “Regulation of membrane repolarization during cardiac muscle cell action potential” and “Negative regulation of voltage-gated potassium channel activity” (Figure 2E), while 3 miRNAs were not previously identified as potential regulator of the resting membrane potential (miR-26b-5p, miR-24-3p, and miR-195-5p). While miR-328-3p and miR-133a-3p were not predicted to target *KCNJ2*, both were predicted to regulate the resting membrane potential via targeting of another member of the Kir2.x family: *KCNJ12* (potassium inwardly rectifying channel subfamily J member 12, Kir2.2).

To summarize, we narrowed down the list of EAT secretome–derived EVs miRNAs to 7 candidates based on predicted regulation of the resting membrane potential (Figure 2E): miR-26a-5p, miR-1-3p, miR-26b-5p, miR-24-3p, miR-328-3p, miR-195-5p, and miR-133a-3p.

### Validation and differential expression of selected miRNAs candidates in EAT vs SAT

**Figure 3:**
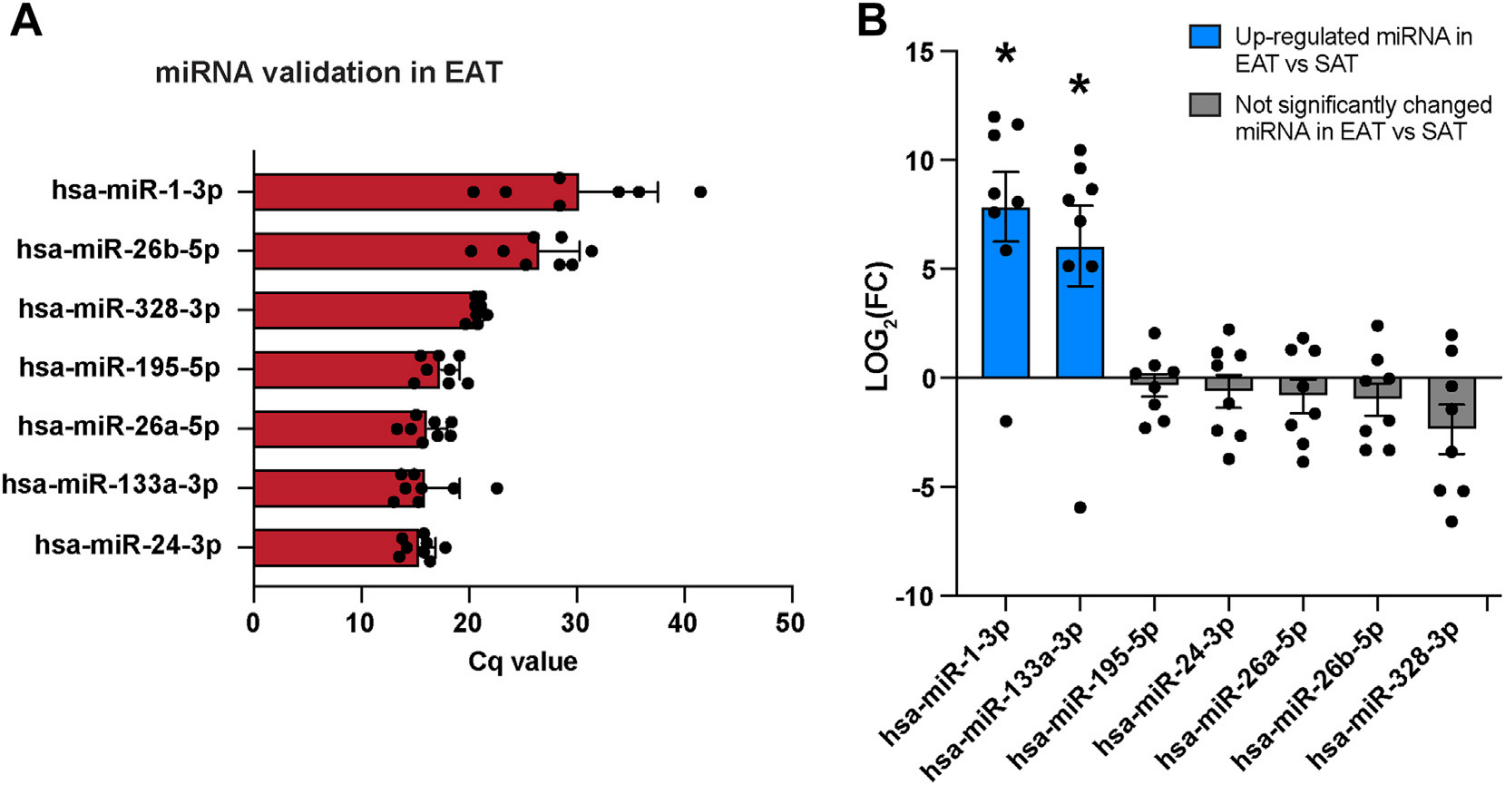
Validation and differential expression of selected microRNAs (miRNAs) in epicardial adipose tissue (EAT) vs subcutaneous adipose tissue (SAT). **A:** Quantification cycle (C_q_) value of the 7 selected miRNA (Figure 3) in EAT isolated from 8 patients. The C_q_ value corresponds to the number of cycles needed for the fluorescence to reach a quantification threshold. **B:** Log_2_ fold change (FC) relative expression of the 7 selected miRNA in EAT vs SAT from 8 paired patient samples. Data are mean ± SEM, Wilcoxon matched-pairs signed rank test. Significantly upregulated miRNA in EAT vs SAT are indicated with blue barplots. Not significantly changed miRNA in EAT vs SAT are indicated with gray barplots. ∗*P <* .05.

Next, we aimed to validate the 7 miRNA candidates previously identified by sequencing in EAT secretome–derived EVs (Figure 2). Due to limited amount of RNA isolated from EVs and the limited amount of EVs in the secretome, we performed this validation in tissue. We collected EAT from 8 patients (patients characteristics provided in Supplemental Table 1) and measured the expression of these miRNAs by quantitative real-time polymerase chain reaction (qRT-PCR). All 7 miRNA candidates identified by sequencing in EAT secretome–derived EVs were expressed in EAT (Figure 3A).

As we previously observed that SAT secretome did not lead to conduction changes or depolarization of the resting membrane or reduction of *Kcnj2* expression, we next compared the expression of the 7 miRNA candidates between EAT and SAT (Figure 3B), assuming that miRNA expression in tissue would correlate with miRNA expression in the secretome. miR-1-3p and miR-133a-3p were significantly upregulated in EAT in comparison with SAT (*P < .*05, n = 8). Specifically, miR-1-3p expression was increased 229.13-fold in EAT compared with SAT. miR-133a-3p expression was increased 65.8-fold in EAT compared with SAT (Figure 3B). miR-195-5p, miR-24-3p, miR-26a-5p, miR-26b-5p, and miR-328-3p expression was not significantly different between EAT and SAT (Figure 3B).

### Transfection of miR-1-3p and miR-133a-3p is arrhythmogenic in NRVMs

**Figure 4:**
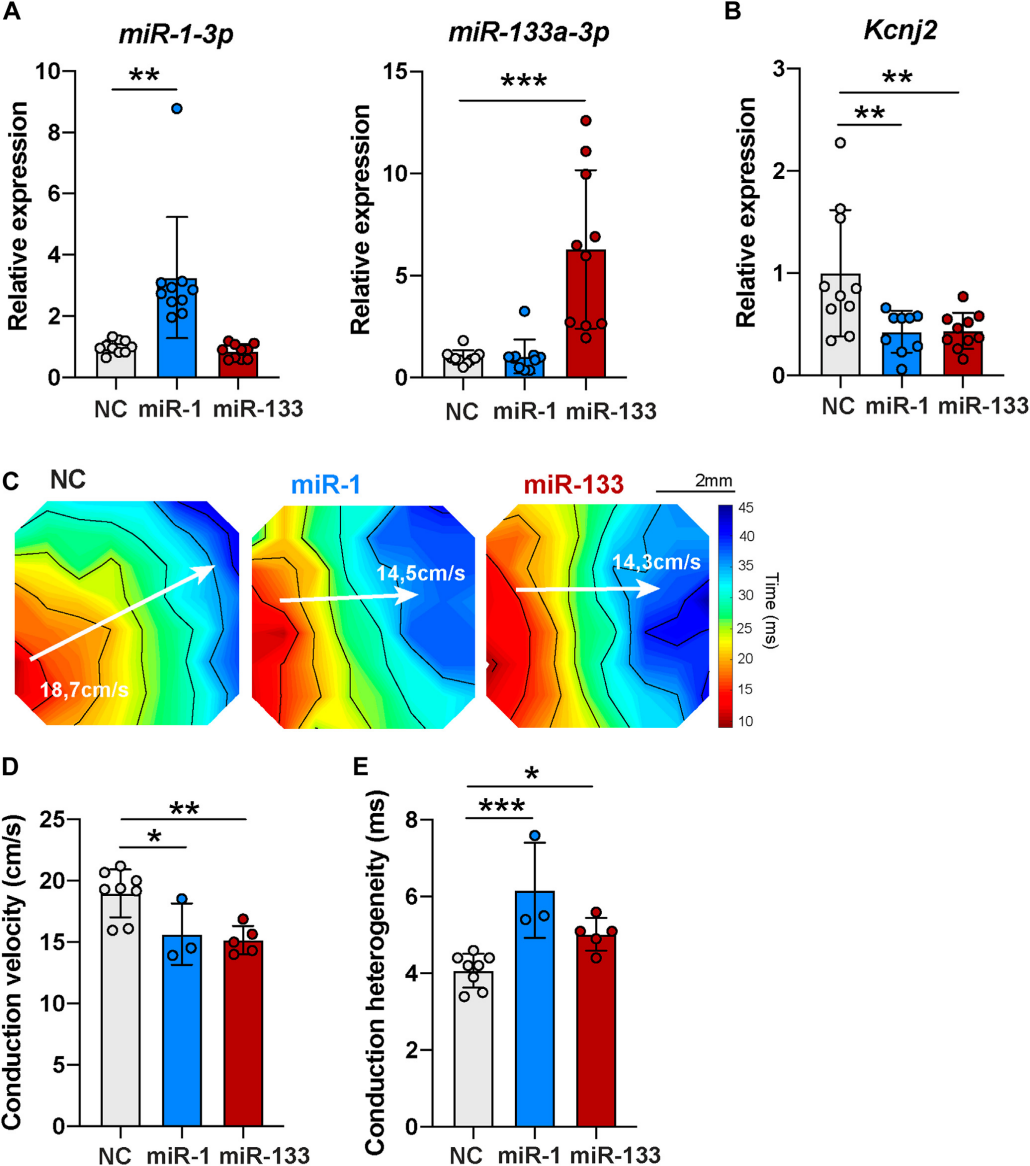
miR-1-3p and miR-133a-3p transfection in neonatal rat ventricular myocytes (NRVMs) leads to arrhythmogenic features. **A:** Relative expression of miR-1-3p and miR-133a-3p in NRVMs transfected with negative control (NC), miR-1-3p and miR-133a-3p, measured by real-time quantitative polymerase chain reaction. Data are mean ± SD. Negative control (NC): n = 10; miR-1-3p: n = 10; miR-133a-3p: n = 10 from 3 independent NRVM isolations. Ordinary 1-way analysis of variance (ANOVA) followed by Dunnett's multiple comparisons test for miR-1-3p. Kruskal-Wallis followed by Dunnett's multiple comparisons test for miR-133a-3p. Primer sequences are included in Supplemental Tables 3 and 4. **B:** Relative expression of *Kcnj2*, gene encoding for Kir2.1, in NRVMs transfected with NC, miR-1-3p, and miR-133a-3p groups, measured by real-time quantitative polymerase chain reaction. Data are mean ± SD. NC: n = 10; miR-1-3p: n = 9; miR-133a-3p: n = 10 from 3 independent NRVM isolation. Ordinary 1-way ANOVA followed by Dunnett's multiple comparisons test. Outlier test (ROUT Q = 1%) identified 1 outlier point in miR-1-3p group that was removed. Primer sequences are included in Supplemental Tables 3 and 4. **C:** Representative activation maps obtained after electrical mapping of NRVMs transfected with NC, miR-1-3p, or miR-133a-3p. Colors indicate activation times according to the scale at right. Isochrones, 5 ms. White arrows indicate where conduction velocity (CV) was measured. CV is indicated for each activation map in white. **D, E:** CV and conduction heterogeneity in paced cardiac monolayers after transfection with NC, miR-1-3p, or miR-133a-3p. Data are mean ± SD, n ≥ 3 monolayers from 1 NRVM isolation. Ordinary 1-way ANOVA followed by Dunnett's multiple comparisons test. ∗*P <* .05, ∗∗*P <* .01, ∗∗∗*P <* .001.

In order to understand the contribution of miR-1-3p and miR-133a-3p in EAT secretome–induced arrhythmogenicity, we transfected NRVMs with miRNA mimics of miR-1-3p and miR-133a-3p and negative control (NC). Transfection of NRVMs with miR-1-3p resulted in a significant 3.26-fold increase of miR-1-3p expression in comparison with NC (fold change [FC] = 3.26 ± 1.98, *P < .*01, n = 10) and did not significantly change the expression of miR-133a-3p (FC = 0.85 ± 0.24, n = 10) (Figure 4A, left). Similarly, miR-133a-3p transfection resulted in a significant 6.29-fold increase of miR-133a-3p expression in NRVMs in comparison with NC (FC = 6.29 ± 3.88, *P < .*001, n = 10) and did not induce significant changes of miR-1-3p expression (FC = 1.05 ± 0.82, n = 10) (Figure 4A, right).

Next, we looked at the consequences of increased miR-1-3p or miR-133a-3p expression on *Kcnj2* expression, conduction velocity and conduction heterogeneity. NRVMs transfected with miR-1-3p or miR-133a-3p mimics showed a significant reduction of *Kcnj2* expression in comparison with NC (FC = 0.43 ± 0.20 [miR-1-3p, n = 9] and FC = 0.44 ± 0.18 [miR-133a-3p, n = 10], *P < .*01) (Figure 4B). In addition, NRVMs transfected with miR-133a-3p showed a significant reduction of *Kcnj12* expression in comparison with NC (FC = 0.56 ± 0.37 [miR-133a-3p, n = 10], *P < .*05) (Supplemental Figure 2).

Potassium ion channels *Kcnj11* and *Kcnq1* expressions were not significantly different between miR-1-3p, miR-133a-3p and NC groups (Supplemental Figure 3; primer sequence in Supplemental Tables 3 and 4). *Scn5a* expression was not significantly different between miR-1-3p and NC groups on the one hand and between miR-133a-3p and NC groups on the other hand. However, the miR-1-3p group showed significantly higher expression of *Scn5a* than miR-133a-3p (FC = 1.17 ± 0.22 vs FC = 0.91 ± 0.24). The subunit *Scn1b* was significantly upregulated with miR-1-3p (FC = 1.36 ± 0.27) and miR-133a-3p (FC = 1.29 ± 0.30) in comparison with NC. *Scn2b* and *Scn3b* expression was not significantly different between the 3 groups. Other pumps and exchanger-encoding genes (*Slc9a1*, *Atp1a1*, *Atp1a2*), as well as calcium-related genes (*Cacna1c*, *Cacna1g*) were not differentially expressed between miR-1-3p, miR-133a-3p, and NC groups.

Figure 4C shows activation maps obtained by electrical mapping of paced cardiomyocytes monolayers 48 hours after transfection with NC, miR-1-3p, or miR-133a-3p. These maps revealed heterogeneous isochrone crowding, indicating a slower conduction in miR-1-3p– and miR-133a-3p–transfected NRVMs than in NC. Overall, conduction velocity was 18.96 ± 1.94, 15.64 ± 2.5, and 15.15 ± 1.15 cm/s in the NC, miR-1-3p, and miR-133a-3p groups, respectively (NC vs miR-1-3p: *P < .*05, n ≥ 3; and NC vs miR-133a-3p: *P < .*01, n ≥ 5) (Figure 4D). Moreover, conduction heterogeneity was 4.07 ± 0.44, 6.17 ± 1.24, and 5.02 ± 0.43 ms in the NC, miR-1-3p, and miR-133a-3p groups, respectively (NC vs miR-1-3p: *P < .*001, n ≥ 3; and NC vs miR-133a-3p: *P < .*05, n ≥ 5) (Figure 4E).

## Discussion

The main finding of our study is the identification of EAT secretome–derived miRNAs as potential mediators of arrhythmogenicity that are transported by EVs. In this study, we show that the EAT secretome contains higher EV concentrations than the SAT secretome. We demonstrate that the EAT secretome–derived EVs contain hundreds of miRNAs, some of them related to cardiac electrophysiology through predicted regulation of resting membrane potential and potassium channels activity. We report that miR-1-3p and miR-133a-3p are more expressed in EAT than in SAT. Finally, we show that overexpression of miR-1-3p or miR-133a-3p in NRVMs recapitulates the conduction slowing effect of the EAT secretome, in association with decreased expression of *Kcnj2* or *Kcnj12*. Thus, the miRNA cargo of EVs from the EAT secretome can be an important player of EAT secretome–induced arrhythmogenicity, and may become a target for therapy.

We previously showed that the EAT secretome but not the SAT secretome leads to arrhythmogenic electrical remodeling of NRVMs.^5^ One of the potential explanations of this difference is that EAT and SAT are different types of body adipose tissue. EAT is a visceral adipose tissue and is considered more metabolically active than subcutaneous fat. Visceral adipose tissue volume is a stronger predictor of metabolic syndrome,^13^ cardiovascular diseases,^14^ and cardiac arrhythmias^15^ than SAT volume.^16^ The mechanisms underlying this difference are not yet fully understood. Distinct adipose tissue depots could secrete different numbers of EVs, acting as local regulators of physiological and pathological pathways. Therefore, we hypothesized that qualitative and quantitative differences in EV secretion between EAT and SAT result in differences in the secretomes and explain their different electrophysiological effects on NRVMs. Our study confirmed this hypothesis and shows that the EV concentration in the EAT secretome is up to 31 times larger than the SAT secretome. To our knowledge, this is the first study to quantify concentrations of EVs released by EAT and SAT. These results are in line with previous work comparing EVs between visceral adipose tissue (from the hypogastric region and the omentum) and SAT of human explants, in which a 35% and a 54% increase in EV concentrations were observed in visceral adipose tissue in comparison with SAT.^17^^,^^18^ We surmise that EVs released by EAT participate in electrical remodeling of NRVMs.

Recently, adipose tissue has been recognized as one of the most important sources of circulating exosomal miRNAs, which can regulate gene expression even in remote tissues.^19^ Considering the proximity between EAT and the myocardium, we speculate that EV-associated miRNAs secreted by EAT are likely to be taken up by cardiomyocytes and exert diverse pathophysiological functions. Using unbiased miRNA sequencing, we deliver, for the first time, an overview of the miRNA profile present in EVs isolated from AF patients and its corresponding validation in tissue. Among the 824 miRNAs identified, only 19% were detected in all 8 patients with EAT secretome–derived EVs, reflecting the diversity of miRNA profiles between patients. The enrichment in extracellular exosomes (EVs from endosomal origin) and EV-related terms in the GO analysis category “cellular component” is consistent with the fact that we sequenced miRNAs encapsulated in EVs.

Of the 7 miRNA candidates selected based on the basis of their potential participation in electrical remodeling of cardiomyocytes, all were present in EAT, and we found that miR-1-3p and miR-133a-3p were increased in EAT in comparison with SAT. We were not able to investigate the miRNA content of SAT-derived EVs due to the low number of EVs derived from the SAT secretome. Thus, we cannot exclude that the miRNA content of EVs may differ between EAT and SAT. In addition, miRNA tissue expression may not completely reflect the miRNA levels detected in EVs.

While miR-1-3p and miR-133a-3p are usually recognized as muscle-enriched miRNAs, they are also expressed in brown adipocytes, while being absent in white adipocytes.^20^ EAT is a white type of adipose tissue exhibiting brown-like adipose tissue features.^21^^,^^22^ Indeed, EAT expresses uncoupling protein 1,^21^ the main marker of brown adipose tissue, and contains small multilocular adipocytes.^23^ The detection of miR-1-3p and miR-133a-3p in this white adipose tissue is another example of its brown fat–like features.

We previously showed that the EAT secretome induces electrophysiological remodeling of cardiomyocytes by reducing *Kcnj2* expression and reducing the inward rectifier K^+^ current. This results in depolarization of the resting membrane of NRVMs, conduction slowing, and increased conduction heterogeneity.^5^ Here, we show that EAT-derived EVs contain miR-1-3p and miR-133a-3p, whose overexpression reduces *Kcnj2* and *Kcnj12* expression, respectively, and exert similar effects on conduction as EAT secretome in NRVMs. miR-1-3p has been shown to target *KCNJ2* and to depolarize the resting membrane of cardiomyocytes,^24^ which is consistent with our results.

To understand the contribution of miRNAs detected in EAT secretome–derived EVs in arrhythmogenesis, we overexpressed miR-1-3p and miR-133a-3p in NRVMs and assessed their effect on conduction. We show that addition of either miRNA decreases conduction velocity and increases conduction heterogeneity, which demonstrates the arrhythmogenic potential of miR-1-3p and miR-133a-3p. Conduction slowing is a main determinant of re-entrant arrhythmias such as AF.^25^ Conduction slowing is arrhythmogenic by reducing wavelength, which increases the risk of re-entry.^26^ Using a computational study, we previously showed that the molecular changes driven by the EAT secretome (conduction slowing and altered electrical coupling) induce sustained re-entrant arrhythmias when EAT covers up to 75% of the epicardium of a human left atrial model.^4^ These results are in line with previous research showing that miR-1-3p overexpression is arrhythmogenic in rat hearts, via conduction slowing and depolarization of the resting membrane by posttranscriptionally repressing *Kcnj2*.^24^

We also show that the 3′ UTR binding site of miR-1-3p on *Kcnj2* is conserved between rat and human (Supplemental Figure 4). This indicates that miR-1-3p from human EAT secretome–derived EVs can target rat *Kcnj2* messenger RNA. On the other hand, miR-133a-3p was not predicted to target *Kcnj2.* However, its overexpression led to a reduction of both *Kcnj12* and *Kcnj2* expression in NRVMs. Indeed, miR-133a-3p was predicted to regulate the resting membrane potential of cardiomyocytes and target Kir2.2 (*Kcnj12*), another isoform of the inward rectifier potassium channel. This is consistent with the fact that the 3′ UTR binding site of miR-133a-3p on *Kcnj12* is conserved between rat and human (Supplemental Figure 5). We speculate therefore that the downregulation of *Kcnj2* is a secondary consequence of miR-133a-3p binding to *Kcnj12*.

The PREDICT-AF (PREventive left atrial appenDage resection for the predICtion of fuTure atrial fibrillation) study^27^ reports that atrial remodeling occurs long before incident AF, and indicates that *KNCJ2* is downregulated in patients who will later develop AF. Based on our results, we speculate that miR-1-3p and miR-133a-3p secreted by EAT facilitate the incidence of AF by early downregulation of *KNCJ2*.

### Study limitations

The EAT and SAT biopsies were obtained from patients presenting multiple age-dependent comorbidities such as overweight, hypertension, diabetes, and heart failure. Arrhythmogenic factors associated with those comorbidities may have contributed to the release of EAT-derived EVs and their miRNAs. However, we selected only the miRNAs that were commonly present in all patients (Figure 2), thereby excluding that only these comorbidities are responsible for the miRNA secretion.

In this study, the EAT and SAT biopsies were obtained exclusively from patients with AF. This exclusivity can be a limitation since it precludes a direct comparison between AF and non-AF donors. The unavailability of tissue samples from non-AF donors restricted our ability to perform this analysis. Nevertheless, our previous findings have indicated that the EAT secretome from non-AF donors can similarly slow conduction, contrary to the idea that observed effects are exclusive to AF patients. Further research is required to ascertain whether the exact role and the quantity of EAT on the heart influences arrhythmic vulnerability and if the content of EVs secreted by EAT evolves with the progression of AF. Our simulation studies suggest that the quantity of EAT of importance for the induction of re-entry.^5^

Further research is required to ascertain whether the exact role and quantity of EAT on the heart influences arrhythmic vulnerability and if the content of EVs secreted by EAT evolves with the progression of AF.

Our study employed a neonatal rat ventricular cardiomyocyte model. In this particular model, the expression of *KCNJ4* (Kir2.3) is low. We acknowledge the importance of exploring the role of Kir2.3 in the context of AF and structural heart disease.

We did not notice any apparent difference in cell viability or morphological abnormalities when comparing miRNA-overexpressing cells with the siNC group. However, we did not perform explicit quantitative analysis to assess this aspect, and therefore we cannot entirely exclude that overexpression of miRNAs caused toxic changes to the cells.

### Clinical implications

Obesity is associated with EAT volume and an increased risk of cardiac arrhythmias.^28^ The EAT secretome facilitates re-entrant arrhythmias by myocardial remodeling.^5^ Understanding the various mechanisms of EAT arrhythmogenicity is critical to prevent its arrhythmogenic effects.

Here, we show that EAT releases EVs containing miRNAs that exert arrhythmogenic effects when the miRNAs are overexpressed in NRVMs.

EVs are recognized as important mediators of intercellular communication.^29^ Circulating EVs can be isolated from blood plasma and therefore are potential biomarkers of cardiovascular diseases.^30^ Because of the physical proximity between EAT and the coronary circulation, EAT-derived EVs could enter the circulation and be detected in blood plasma. Quantification of EAT-derived EVs concentration in blood plasma may provide a surrogate measure of epicardial adipose tissue volume and AF risk. Further research is necessary to identify specific markers of EAT-derived EVs.

Current treatments of atrial and ventricular arrhythmias using antiarrhythmic drug therapy show limited effectiveness^31^ and often result in adverse reactions because of a lack of tissue selectivity. This study provides new insights on the mechanisms of arrhythmogenesis and give potential inroads for the development of novel therapeutic targets of arrhythmias.

Adipose tissue is a substantial source of circulating miRNAs contained in EVs.^19^ In this study, we show that EVs derived from EAT contain miR-1-3p and miR-133a-3p, which exert arrhythmogenic effects when overexpressed in NRVMs. EAT-targeted administration of anti-miRNA antisense oligomer using viral vectors is a potential strategy to decrease the expression of those arrhythmogenic miRNAs. However, the efficiency of this approach is limited by potential off target effects, lack of viral vectors specific for EAT, and challenges in vector administration route. Therapeutical strategies based on biologic response modifiers might exert higher success rates.

Reduction of EAT volume could decrease EVs release and subsequently reduce the levels of arrhythmogenic miRNAs. Thus, reduction of EAT volume is a potential therapeutical strategy to reduce cardiovascular risk. Systematic reviews and meta-analyses have shown that exercise, hypocaloric diet, bariatric surgery, and pharmaceutical interventions (lipid-lowering and antidiabetic therapies) are effective means to reduce EAT volume.^32^ Therefore, patients with excessive EAT volume can benefit from these novel therapeutic strategies targeting EAT secretome content.

## Conclusion

miR-1-3p and miR-133a-3p, carried by EVs, are potential mediators of EAT arrhythmogenicity and form a possible target for antiarrhythmic therapy.
