# Supplementary material for: MicroRNAs in extracellular vesicles released from epicardial adipose tissue promote arrhythmogenic conduction slowing
Source: Heart Rhythm O2. 2023 Nov 2;4(12):805–14. doi: 10.1016/j.hroo.2023.10.007 (PMC10774655; doi:10.1016/j.hroo.2023.10.007)
Supplement: Supplemental Tables and Figures [file mmc1.docx]

**Supplemental Appendix**

**Supplemental Methods**

**Human adipose tissue collection**

17 patients with symptomatic, persistent or long-standing persistent AF undergoing video-assisted thoracoscopic pulmonary vein isolation were recruited at the Amsterdam UMC, University of Amsterdam, between 2019 and 2022. Written informed consent was obtained from all participants^1^. The study was conducted in accordance with the Declaration of Helsinki as revised in 2013 and with the Medical Research Involving Human Subjects Act (WMO) and was approved by the Institutional Review Board of the Amsterdam AMC. AF was documented on ECG, Holter or pacemaker electrogram at least once in the 12 months preceding surgery. Patients with symptoms of severe heart failure or a left ventricular ejection fraction <35% were excluded from the study. The clinical characteristics of patients are listed in **Supplemental Table 1**. Patient EAT and SAT samples or the secretome derived from it were used for flow cytometry, miRNA sequencing and/or miRNA validation experiments (**Supplemental** **Table 1**).

During thoracoscopic ablation the left atrial appendage (LAA) was resected as standard procedure. The atrial tissue was collected and the EAT overlying the LAA was removed. A biopsy of approximately 1 cm^3^ of SAT was obtained from one of the thoracoscopic entry sites. Adipose tissue was transported in phosphate-buffered saline to the laboratory for secretome extraction, or immediately snap-frozen for subsequent RNA isolation.

**Adipose tissue secretome collection**

The method for secretome collection has been described previously^5^. Briefly, EAT and SAT were rinsed and minced into 1-2 mm^3^ explants. Each piece was transferred to a well of a 96-well plate and incubated in 100 μL culture medium for 24 hours at 37°C and 5% CO_2_ in order to stimulate adipokine and secretome release^2^. EAT and SAT secretome samples from each patient were collected, pooled, filtered (0.45 µm pore size hydrophilic PVDF membrane, Millipore, SLHV033RB) and stored at -80°C. Adipose tissue was weighted after secretome collection to ensure similar adipose tissue weight per mL of secretome (**Supplemental Figure 1**).

### Concentration measurements of extracellular vesicles by flow cytometry

### Flow cytometry (A60-Micro, Apogee Flow Systems, Hemel Hempstead, UK) was used to determine the EV concentration^3^ in adipose tissue secretome samples collected from 7 patients (clinical characteristics provided in Supplemental Table 1) and control culture medium without serum. For each of the 7 patients, we collected EAT and SAT samples, and extracted its secretome.

### To achieve a count rate below 5000 events/seconds upon measuring, thereby preventing swarm detection^4^, adipose tissue conditioned culture medium samples were diluted 1.5 to 2-fold in Dulbecco’s phosphate-buffered saline prior to antibody labelling. To label EVs, 20 μL of diluted sample was incubated with 2.5 μL of antibodies (antibody references are provided in MIFlowCyt-EV supplement) and kept in the dark at room temperature for 2 hours. We labelled EVs using tetraspanin markers (CD63 and CD9), and lactadherin to identify phosphatidylserine exposing EVs. To decrease background fluorescence by unbound reagents, 200 μL of Dulbecco’s phosphate-buffered saline was added to the sample prior to measurement. Samples were measured for 120 s at a flow rate of 3.01 μL/min. The trigger threshold was set to 24 arbitrary units of the side scattering detector (side scattering cross section of 8 nm^2^). The reported concentration of EVs per mL of secretome refers to the number of particles (i) exceeding the side scattering threshold, (ii) having a diameter <1,000 nm, and (iii) exceeding the fluorescent gate corresponding to the used labels.

### To improve the reproducibility, flow cytometry data were reported according to the MIFlowCyt-EV framework^5^. We calibrated all detectors, applied assay controls to confirm that signals originate from EVs, and automated data calibration and processing with custom-built software^6^ (MATLAB R2020b, Mathworks, USA).

**Neonatal rat ventricular myocytes isolation**

All animal experiments were approved by the local Animal Experiments Committee (Academic Medical Center, University of Amsterdam) and carried out in compliance with the Guide for the Care and Use of Laboratory Animals and in accordance with national and institutional guidelines. NRVMs were isolated from 1-to-2-day-old Wistar rats (Janvier labs) as previously described. Pups were anesthetized with isoflurane and hearts were excised after decapitation. Ventricular myocardium was cut into pieces and dissociated with trypsin (1 mg/mL;Sigma), and collagenase type 2 (1 mg/mL;Worthington). Cells were collected and resuspended in TUNG culture medium [M199 medium (Gibco) supplemented with 10% heat inactivated fetal bovine serum (FBS, Gibco), 1% HEPES (Gibco), 5000 U/L penicillin-G (Sigma), 2 mg/L vitamin B12 (Sigma-Aldrich), 3.5 g/L glucose, 1% non-essential amino acids (Gibco), 1% L-glutamine (Gibco)]. The cell suspension was pre-plated for 2 hours to minimize fibroblast contamination. The remaining myocardial cells were plated on microelectrode arrays (MEAs, Multi-Channel Systems MCS GmbH, Reutlingen, Germany) or 6-well-plates coated with fibronectin (Corning, Ref 356008, Bedford, MA, USA) at a density of 1.4x10^5^ cells per cm^2^. NRVMs were cultured at 37°C and 5% CO_2_ for 3 days in TUNG medium.

**Transfection with miRNA mimics**

At 20–24 hours after seeding, NRVMs were transfected with 100 nM negative control siRNA (Qiagen, Ref 1027417, Germantown, MD, USA), 100 nM Syn-hsa-miR-133a-3p miScript miRNA Mimic or 100 nM Syn-hsa-miR-1-3p miScript miRNA Mimic (Qiagen, Ref 219600, Germantown, MD, USA) using Lipofectamine™ RNAiMAX Transfection Reagent (Thermo Fisher Scientific, Ref 13778150, Carlsbad, CA, USA) in M199 medium (Gibco) without supplements. After 6 hours of incubation, cell culture medium was replaced by TUNG with 2% FBS. Cells were harvested or measured at 48 hours after transfection. miRNA mimic sequences are found in **Supplemental** **Table 3**.

**qRT-PCR**

RNA was extracted from EAT, SAT and NRVMs with TRI reagent (Sigma-Aldrich) following manufacturer’s instructions.

Total RNA was converted into cDNA using the qScript microRNA cDNA Synthesis kit (Quantabio, CA, USA) according to the manufacturers' instructions.

Briefly, miRNAs were polyadenylated in a poly(A) polymerase reaction. qScript Reverse Transcriptase and other necessary reagents were added to convert the poly(A) tailed miRNAs and poly(A) containing mRNA into cDNA using an oligo-dT primer with an adapter. This miRNA-based cDNA was amplified in a RT-qPCR reaction with a miRNA-specific forward primer (sequences reported in **Supplemental** **Table 4**) and a general reverse primer binding to the adapter of the oligo-dT primer. RT-qPCR of mRNA was performed with gene-specific intron-spanning forward and reverse primers. RT-qPCR was then performed on a LightCycler 480 (Roche) using the PerfeCTa SYBR Green Kit (Quantabio, USA) and the following program:.

Results were analyzed using LinRegPCR software^7^. For *Kcnj2* gene expression quantification, the geometric mean of the reference genes *Hprt1* and *Eef1e1* was used to normalize expression levels and depicted gene expression levels are shown relative to the control condition in each NRVM isolation. *U6* was used as a reference gene to normalize miRNA expression levels.

**Electrophysiology**

Electrical mapping was performed as previously described. Briefly, 10 minutes before measurements, culture media was replaced by a modified Tyrode’s solution (36.5°C) containing (mM): NaCl 140, KCl 5.4, CaCl_2_ 1.8, MgCl_2_ 1.0, glucose 5.5, HEPES 5.0; pH 7.4 (adjusted with NaOH).

NRVMs cultured on multi-electrode arrays (MEAs, 60 electrodes terminals, 60EcoMEA-Glass-gr, MultiChannel Systems MCS GmbH) were stimulated using a bipolar extracellular stimulus electrode (1.5x diastolic stimulation threshold, 2ms pulse). A reference electrode was placed inside the MEAs, not in contact with the cells. Unipolar electrograms were recorded simultaneously with a 256-channel amplifier (BioSemi, 24 bit dynamic range, 122.07 nV LSB, total noise 0.5 µV). Signals were recorded with a sampling frequency of 2048 Hz (bandwidth (‑3dB) DC–400 Hz).

Data analysis was performed using a custom-made data analysis program written in Matlab 2006b (The MathWorks Inc, Natick, MA^8^). Local activation time was determined as the interval from the stimulation artefact to the minimum derivative of the local QRS, and used to construct activation maps.

Blinded analysis was performed to measure conduction velocity, which was determined along lines perpendicular to isochronal lines by dividing the difference in local activation times by the electrode distance. Three conduction velocity measurements were performed and averaged per monolayer. Heterogeneity in conduction was measured based on the method described by Lammers et al^9^. Heterogeneity in conduction was determined as follows: at every electrode recording location, the maximal difference with neighboring activation times was calculated, known as the phase difference. These local phase differences were then visualized on a phase map, revealing the spatial pattern of heterogeneity in conduction. Subsequently, the median value of heterogeneity was derived from each of these maps.

**EAT secretome-derived EV isolation**

Frozen EAT secretome samples from 8 patients were thawed on ice, and concentrated to 1 mL using Amicon Ultra-15mL Centrifugal Filter Units. The clinical characteristics of patients are listed in **Supplemental** **Table 1**.

EAT secretome-derived extracellular vesicles (EATS-EVs) were isolated using qEVoriginal 70 nm columns (IZON Science LTD) combined with an automatic fraction collector. The EV enriched fractions 2,3 and 4 (between 0.5 and 2 mL of the Purified Collection Volume) were pooled and concentrated to 200 µL using Amicon Ultra-2mL centrifugal Filter Unit with a 10 kDa cut off (Merck#UFC201024). RNA from EATS-EVs was isolated using the miRNeasy serum/plasma kit (QIAgen) according to the manufacturers' protocol.

**Small RNA library preparation and sequencing**

miRNA sequencing was performed by ExBiome as previously described^10^ and as detailed below. Small RNA libraries were prepared using a previously published IsoSeek protocol^10^. Briefly, custom designed adapters were used in combination with the NEBNext Multiplex Small RNA Library Prep Kit for Illumina. The number of PCR amplification cycles was 20. Libraries were quantified with the KAPA Library Quantification Kit (Roche), mixed at equimolar concentration (1.5 nM each) and sequenced on a MiSeq platform (PE50, v2 300 cycles, with the addition of 10% PhiX).

**Processing of sequencing data and miRNA profiling**

Pre-processing and mapping of adapter trimmed reads were performed using the latest version of sRNAbench command line tool^11^. Default parameters were used for all analysis steps after pre-processing and miRBase v22.1^12^ was used as miRNA reference. Quality control of samples was carried out using mirnaQC^13^ to rule out technical differences between libraries.

**Prediction analysis**

GeneTrail2^14^ was used to perform Gene Ontology (GO) analysis on 'cellular component' and 'biological process' terms based on predicted targets. To perform this analysis, the 156 miRNAs identified in all 8 patients were used as input in GeneTrail2. The indicated q-value corresponds to the adjusted *P*-value for multiple testing using the Benjamin-Yekutieli method. The indicated enrichment value corresponds to the number of hits divided by the expected score. TargetScan^15^ was used to predict miRNAs targeting *KCNJ2*, using as input list the 100 most highly expressed miRNAs identified in all 8 patients. In addition, the miRDB^16^ prediction database was used to identify potassium channel targets of miRNAs identified as regulators of resting membrane potential, but not predicted to target *KCNJ2*.

References

1. Krul SP, Driessen AH, van Boven WJ, et al. Thoracoscopic video-assisted pulmonary

vein antrum isolation, ganglionated plexus ablation, and periprocedural

confirmation of ablation lesions: first results of a hybrid surgicalelectrophysiological

approach for atrial fibrillation. Circ Arrhythm Electrophysiol 2011;4:262–270.

2. Viviano A, Yin X, Zampetaki A, et al. Proteomics of the epicardial fat secretome

and its role in post-operative atrial fibrillation. Europace 2018;

20:1201–1208.

3. Eyileten C, Jakubik D, Shahzadi A, et al. Diagnostic performance of circulating

miRNAs and extracellular vesicles in acute ischemic stroke. Int J Mol Sci

2022;23:4530.

4. Buntsma NC, Ga˛secka A, Roos Y, et al. EDTA stabilizes the concentration of

platelet-derived extracellular vesicles during blood collection and handling. Platelets

2022;33:764–771.

5. Welsh JA, Van Der Pol E, Arkesteijn GJA, et al. MIFlowCyt-EV: a framework for

standardized reporting of extracellular vesicle flow cytometry experiments. J Extracell

Vesicles 2020;9:1713526.

6. Gasecka A, Nieuwland R, Budnik M, et al. Ticagrelor attenuates the increase of

extracellular vesicle concentrations in plasma after acute myocardial infarction

compared with clopidogrel. J Thromb Haemost 2020;18:609–623.

7. Ruijter JM, Ramakers C, Hoogaars WM, et al. Amplification efficiency: linking

baseline and bias in the analysis of quantitative PCR data. Nucleic Acids Res

2009;37:e45.

8. PotseM, Linnenbank AC, Grimbergen CA. Software design for analysis of multichannel

intracardial and body surface electrocardiograms. Comput Methods Programs

Biomed 2002;69:225–236.

9. Lammers WJ, Schalij MJ, Kirchhof CJ, Allessie MA. Quantification of spatial inhomogeneity

in conduction and initiation of reentrant atrial arrhythmias. Am J

Physiol 1990;259:H1254–H1263.

10. van Eijndhoven MAJ, Aparicio-Puerta E, Gómez-Martín C, et al. Unbiased and

UMI-informed sequencing of cell-free miRNAs at single-nucleotide resolution.

bioRxiv https://doi.org/10.1101/2021.05.04.442244.

11. Aparicio-Puerta E, Gómez-Martín C, Giannoukakos S, et al. sRNAbench

and sRNAtoolbox 2022 update: accurate miRNA and sncRNA profiling

for model and non-model organisms. Nucleic Acids Res 2022;50:

W710–W717.

12. Kozomara A, Birgaoanu M, Griffiths-Jones S. miRBase: from microRNA sequences

to function. Nucleic Acids Res 2018;47:D155–D162.

13. Aparicio-Puerta E, Gómez-Martín C, Giannoukakos S, et al. mirnaQC: a webserver

for comparative quality control of miRNA-seq data. Nucleic Acids Res

2020;48:W262–W267.

14. Gerstner N, Kehl T, Lenhof K, et al. GeneTrail 3: advanced high-throughput

enrichment analysis. Nucleic Acids Res 2020;48:W515–W520.

15. McGeary SE, Lin KS, Shi CY, et al. The biochemical basis of microRNA targeting

efficacy. Science 2019;366:eaav1741.

16. Chen Y, Wang X. miRDB: an online database for prediction of functional micro-RNA

Targets. Nucleic Acids Res 2020;48:D127-D131.

**Supplemental Figures and Tables**

**
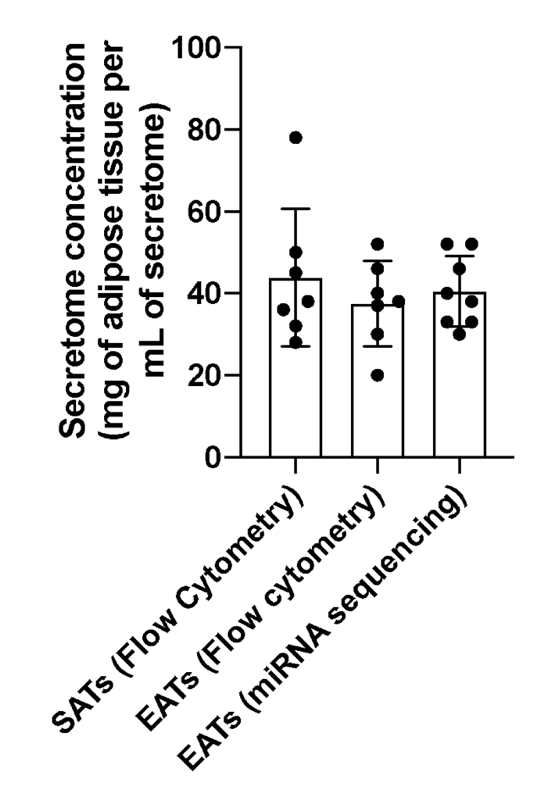
**

**Supplemental Figure 1. Secretome samples concentrations.**

Concentration of EAT and SAT secretome samples used for Flow cytometry or miRNA sequencing experiments expressed in mg of adipose tissue per mL of secretome. There was no significant differences in concentrations between the groups. One-way ANOVA. n=7 minimum.


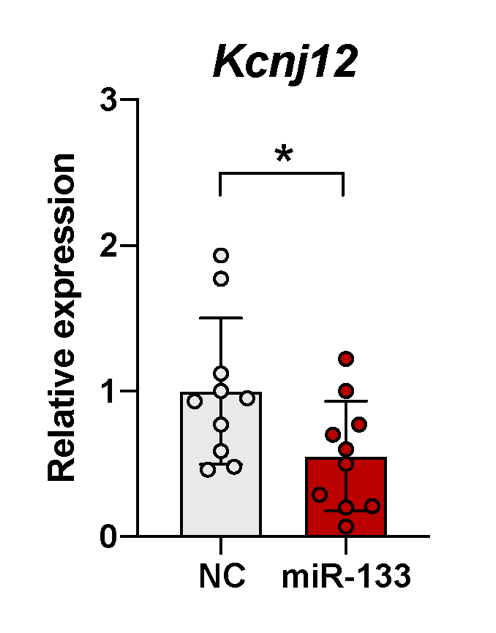


**Supplemental Figure 2. miR-133a-3p overexpression reduces *Kcnj12* expression.**

Relative expression of *Kcnj12*, gene encoding for Kir2.2, in NRVMs transfected with NC and miR‑133a‑3p, measured by real-time qPCR. Data are mean±SD. NC: n=10, miR‑133a‑3p: n=10 from three independent NRVMs isolation. Student's t-test. **P*<.05. Primer sequences are included in Supplemental Table 4.

**
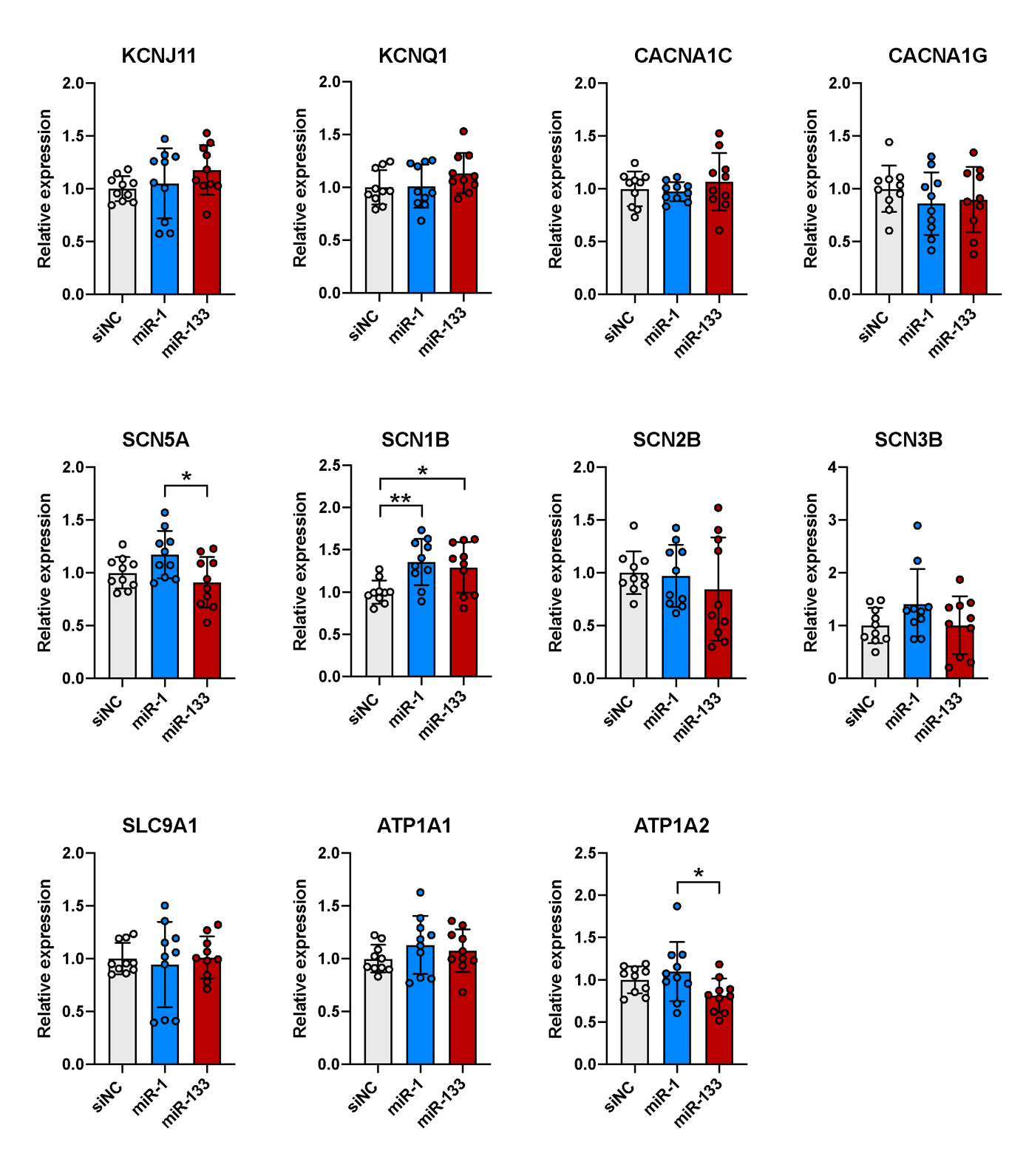
**

**Supplemental Figure 3. Ion channels expression**

Relative expression of *Kcnj11*, *Kcnq1*, *Cacna1c*, *Cacna1g*, *Scn5a*, *Scn1b*, *Scn2b*, *Scn3b*, *Slc9a1*, *Atp1a1* and *Atp1a2* in NRVMs transfected with siNC, miR-1-3p and miR‑133a‑3p, measured by real-time qPCR. Data are mean±SD. siNC: n=10, miR-1-3p: n=10, miR‑133a‑3p: n=10 from three independent NRVMs isolation. One-way ANOVA followed by Tukey's multiple comparisons test in case of normal distribution, or by Dunn's multiple comparison test. *P<.05. **P<.01. Primer sequences are included in Supplemental Table 4.


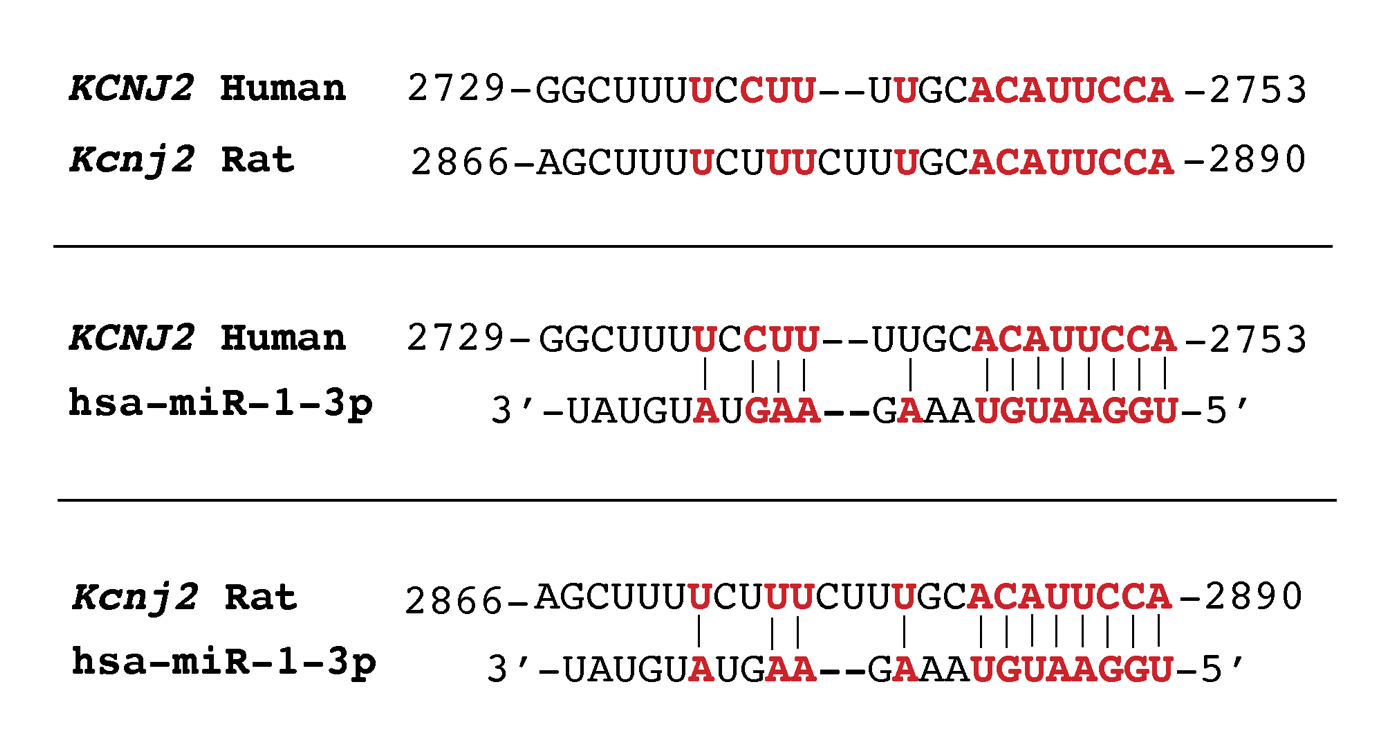


**Supplemental Figure 4. miR-1-3p predicted binding sites on *KCNJ2***

Complementary sequences between miR-1-3p and the predicted 3'UTRs binding sites of *KCNJ2* messenger RNA in human (ENST00000243457.4, ensembl.org) and rat (ENSRNOG00000064933, ensembl.org).


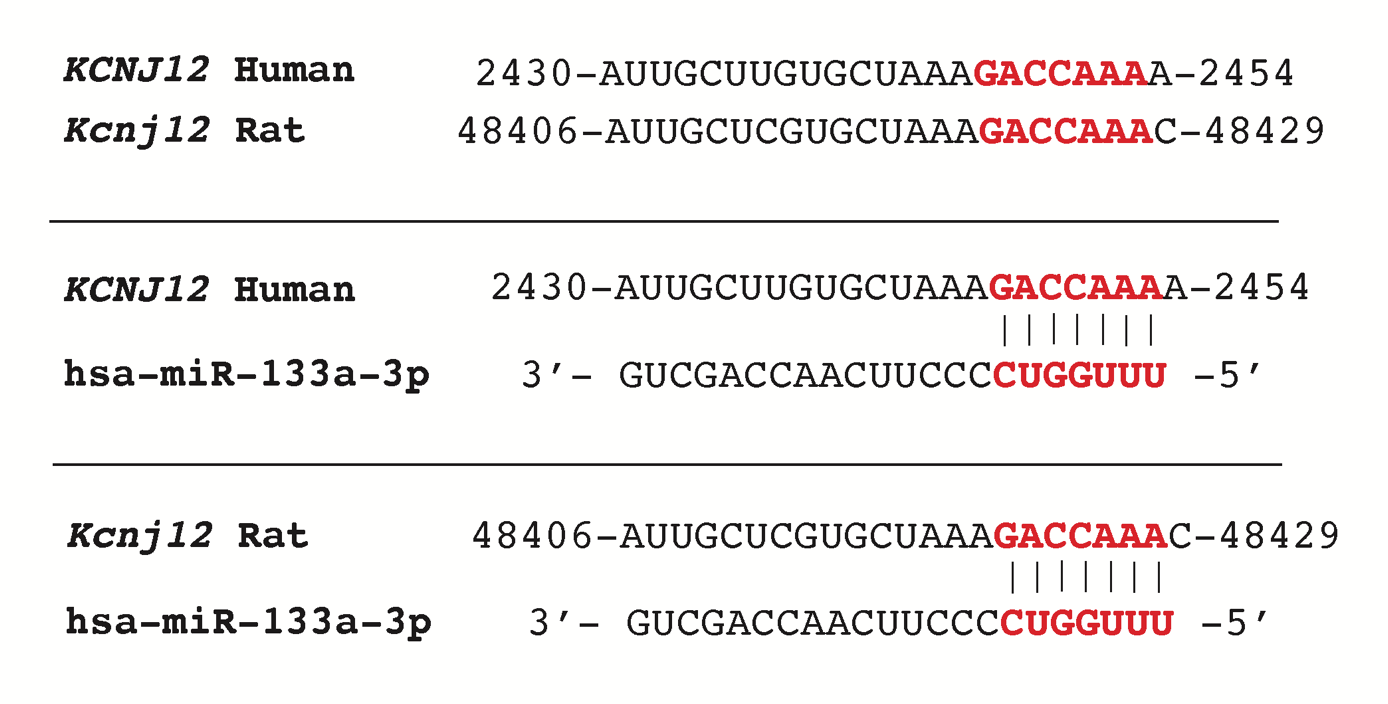


**Supplemental Figure 5. miR-133a-3p predicted binding sites on *KCNJ12***

Complementary sequences between miR-1-3p and the predicted 3'UTRs binding sites of *KCNJ12* messenger RNA in human (ENST00000583088.6, ensembl.org) and rat (ENSRNOG00000002303, ensembl.org). Target sequence identified using targetscan.org.

**Supplemental Table 1. Patient characteristics**

| ***Experiment*** | ***All experiments*** | | ***Flow cytometry (paired EAT and SAT secretome samples)*** | | ***miRNA sequencing*** | | ***miRNA validation in EAT and SAT*** | |
| --- | --- | --- | --- | --- | --- | --- | --- | --- |
| ***Clinical characteristics*** | *n, mean±SD (range)* | *%* | *n, mean±SD (range)* | *%* | *n, mean±SD (range)* | *%* | *n, mean±SD (range)* | *%* |
|  |  |  |  |  |  |  |  |  |
| **Total** | 17 |  | 7 |  | 8 |  | 8 |  |
| **Sex** |  |  |  |  |  |  |  |  |
| Male | 10 | 58.8% | 6 | 85.7% | 5 | 62.5% | 5 | 62.5% |
| Female | 7 | 41.2% | 1 | 14.3% | 3 | 37.5% | 3 | 37.5% |
| **Age** (years) | 63±8  (39-75) |  | 63±5  (57-73) |  | 64±6  (57-75) |  | 61±9  (39-70) |  |
| **Body Mass index** (kg/m^2^) | 28±3  (24-36) |  | 28±2  (25-33) |  | 27±3  (24-34) |  | 29±4  (25-36) |  |
| **Atrial Fibrillation** |  |  |  |  |  |  |  |  |
| Persistent AF | 16 | 94% | 7 | 100% | 8 | 100% | 7 | 87.5% |
| Long-standing persistent AF | 1 | 6% | 0 | 0% | 0 | 0% | 1 | 12.5% |
|  |  |  |  |  |  |  |  |  |
| **Comorbidities** |  |  |  |  |  |  |  |  |
| Hypertension | 10 | 59% | 7 | 100% | 6 | 75% | 3 | 38% |
| Vascular diseases | 0 | 0% | 0 | 0% | 0 | 0% | 0 | 0% |
| Diabetes  Previous stroke  Heart failure  **Age groups**  Age ≥75  Age 65-74  Age <65 | 2  2  4  1  6  10 | 12%  12%  24%  6%  35%  59% | 0  1  1  0  2  5 | 0%  14%  14%  0%  29%  71% | 2  1  3  1  2  5 | 25%  13%  38%  13%  25%  63% | 0  0  1  0  3  5 | 0%  0%  13%  0%  38%  63% |

**Supplemental Table 2**

| **miRNA** | **Averaged read-counts** |
| --- | --- |
| hsa-let-7b-5p | 231807 |
| hsa-let-7a-5p | 166820 |
| hsa-let-7c-5p | 138335 |
| hsa-miR-26a-5p | 52645 |
| hsa-let-7f-5p | 43885 |
| hsa-miR-99a-5p | 32951 |
| hsa-miR-125b-5p | 23020 |
| hsa-miR-100-5p | 17556 |
| hsa-miR-320a-3p | 16617 |
| hsa-miR-143-3p | 13779 |
| hsa-miR-10b-5p | 13681 |
| hsa-miR-92a-3p | 13441 |
| hsa-let-7e-5p | 12678 |
| hsa-miR-30d-5p | 11607 |
| hsa-miR-148a-3p | 9971 |
| hsa-miR-125a-5p | 8556 |
| hsa-miR-1-3p | 8378 |
| hsa-miR-26b-5p | 7208 |
| hsa-miR-10a-5p | 7020 |
| hsa-miR-199a-3p | 6766 |
| hsa-miR-21-5p | 6148 |
| hsa-miR-23a-3p | 5949 |
| hsa-miR-101-3p | 5640 |
| hsa-miR-193a-5p | 5586 |
| hsa-miR-99b-5p | 5543 |
| hsa-miR-25-3p | 5520 |
| hsa-miR-486-5p | 5268 |
| hsa-miR-423-5p | 5046 |
| hsa-let-7i-5p | 4379 |
| hsa-miR-146b-5p | 4239 |
| hsa-miR-151a-3p | 3970 |
| hsa-miR-152-3p | 3774 |
| hsa-miR-574-5p | 3770 |
| hsa-miR-214-3p | 3738 |
| hsa-let-7d-5p | 3700 |
| hsa-miR-199b-3p | 3347 |
| hsa-miR-92b-3p | 3300 |
| hsa-miR-30a-5p | 3079 |
| hsa-miR-218-5p | 3068 |
| hsa-let-7g-5p | 2893 |
| hsa-miR-28-3p | 2540 |
| hsa-miR-222-3p | 2462 |
| hsa-miR-191-5p | 2087 |
| hsa-miR-127-3p | 2060 |
| hsa-miR-30e-3p | 2035 |
| hsa-miR-532-5p | 1795 |
| hsa-miR-3960 | 1463 |
| hsa-miR-30e-5p | 1453 |
| hsa-miR-423-3p | 1316 |
| hsa-miR-342-3p | 1300 |
| hsa-miR-23b-3p | 1249 |
| hsa-miR-30a-3p | 1196 |
| hsa-miR-140-3p | 1102 |
| hsa-miR-24-3p | 1095 |
| hsa-let-7d-3p | 1094 |
| hsa-miR-451a | 1092 |
| hsa-miR-27a-3p | 1079 |
| hsa-miR-30c-5p | 1078 |
| hsa-miR-150-5p | 1055 |
| hsa-miR-103a-3p | 1041 |
| hsa-miR-574-3p | 956 |
| hsa-miR-27b-3p | 949 |
| hsa-miR-98-5p | 915 |
| hsa-miR-29a-3p | 882 |
| hsa-miR-328-3p | 811 |
| hsa-miR-432-5p | 784 |
| hsa-miR-181a-5p | 760 |
| hsa-miR-378a-3p | 734 |
| hsa-miR-1246 | 699 |
| hsa-miR-374b-5p | 693 |
| hsa-miR-139-5p | 665 |
| hsa-miR-148b-3p | 657 |
| hsa-miR-126-3p | 651 |
| hsa-miR-184 | 633 |
| hsa-miR-221-3p | 629 |
| hsa-miR-361-3p | 628 |
| hsa-miR-4510 | 616 |
| hsa-miR-374a-5p | 611 |
| hsa-miR-195-5p | 606 |
| hsa-miR-199b-5p | 576 |
| hsa-miR-382-5p | 566 |
| hsa-miR-181b-5p | 562 |
| hsa-miR-1307-3p | 559 |
| hsa-miR-155-5p | 544 |
| hsa-miR-320b | 520 |
| hsa-miR-409-3p | 507 |
| hsa-miR-224-5p | 500 |
| hsa-miR-133a-3p | 489 |
| hsa-miR-22-3p | 483 |
| hsa-miR-126-5p | 470 |
| hsa-miR-125b-2-3p | 460 |
| hsa-miR-197-3p | 442 |
| hsa-miR-335-3p | 432 |
| hsa-miR-193b-5p | 424 |
| hsa-miR-379-5p | 417 |
| hsa-miR-4516 | 386 |
| hsa-miR-186-5p | 385 |
| hsa-miR-4488 | 382 |
| hsa-miR-93-5p | 381 |
| hsa-miR-361-5p | 357 |
| hsa-miR-485-5p | 350 |
| hsa-miR-204-5p | 349 |
| hsa-miR-146a-5p | 340 |
| hsa-miR-30b-5p | 335 |
| hsa-miR-483-5p | 328 |
| hsa-miR-132-3p | 312 |
| hsa-miR-151a-5p | 307 |
| hsa-miR-128-3p | 302 |
| hsa-miR-223-3p | 290 |
| hsa-miR-484 | 285 |
| hsa-miR-199a-5p | 283 |
| hsa-miR-34a-5p | 258 |
| hsa-miR-1180-3p | 240 |
| hsa-miR-134-5p | 238 |
| hsa-miR-190a-5p | 235 |
| hsa-miR-320c | 234 |
| hsa-let-7b-3p | 233 |
| hsa-miR-744-5p | 233 |
| hsa-miR-106b-3p | 226 |
| hsa-miR-203a-3p | 217 |
| hsa-miR-1260a | 215 |
| hsa-miR-99b-3p | 204 |
| hsa-miR-4454 | 204 |
| hsa-miR-16-5p | 200 |
| hsa-miR-874-3p | 195 |
| hsa-miR-320d | 195 |
| hsa-miR-1247-5p | 184 |
| hsa-miR-2110 | 175 |
| hsa-miR-139-3p | 173 |
| hsa-miR-135a-5p | 171 |
| hsa-miR-543 | 165 |
| hsa-miR-92b-5p | 157 |
| hsa-miR-1260b | 155 |
| hsa-miR-342-5p | 154 |
| hsa-miR-7704 | 150 |
| hsa-miR-340-3p | 144 |
| hsa-miR-452-5p | 143 |
| hsa-miR-660-5p | 143 |
| hsa-miR-505-3p | 138 |
| hsa-miR-671-5p | 127 |
| hsa-miR-365b-3p | 121 |
| hsa-miR-365a-3p | 121 |
| hsa-miR-369-5p | 118 |
| hsa-miR-1301-3p | 109 |
| hsa-miR-340-5p | 109 |
| hsa-miR-433-3p | 106 |
| hsa-miR-877-5p | 101 |
| hsa-miR-181d-5p | 100 |
| hsa-miR-365a-5p | 100 |
| hsa-miR-185-5p | 99 |
| hsa-miR-193b-3p | 90 |
| hsa-miR-425-5p | 80 |
| hsa-miR-374a-3p | 67 |
| hsa-miR-145-3p | 66 |
| hsa-miR-1290 | 63 |
| hsa-miR-1306-5p | 53 |

**Supplemental Table 3. miRNA mimic sequences**

| **miRNA mimic** | **Sequence 5'-3'** |
| --- | --- |
| hsa-miR-1-3p | UGGAAUGUAAAGAAGUAUGUAU |
| hsa-miR-133a-3p | UUUGGUCCCCUUCAACCAGCUG |

**Supplemental Table 4. Primer sequences**

| **Primer** | **Sequence** |
| --- | --- |
| Atp1a1 Fw | CGGATGACAGTGGCTCACAT |
| Atp1a1 Rv | CGCTACTGCACGCTTAAGGA |
| Atp1a2 Fw | CATCATTTGCAAGACCCGGC |
| Atp1a2 Rv | AACCACCACGTGACCTTGAG |
| Cacna1c Fw | TGGGATCATGGCTTATGGCGGC |
| Cacna1c Rv | ATCAGCCAGGTTGTCCACCG |
| Cacna1g Fw | AGGCAGAGGAAATCGGCAAA |
| Cacna1g Rv | CTGTCCCCATCACCATCCAC |
| Eef1e1 Fw | TCCAGTAAAGAAGACACCCAGA |
| Eef1e1 Rv | GACAAAACCAGCGAGACACA |
| Gapdh Fw | GGTGGACCTCATGGCCTACA |
| Gapdh Rv | CTCTCTTGCTCTCAGTATCCTTGCT |
| Hprt Fw | TGACTATAATGAGCACTTCAGGGATTT |
| Hprt Rv | CGCTGTCTTTTAGGCTTTGTACTTG |
| Kcnj2 Fw | TGTGTTACAGACGAGTGCCC |
| Kcnj2 Rv | CAGAGTTTGCCGTCCCTCAT |
| Kcnj11 Fw | ATCAGTCCAGAGGTTGGTGC |
| Kcnj11 Rv | TAATGCCCTTTCGGGACAGC |
| Kcnj12 Fw | AACCCCTACAGCATCGTATC |
| Kcnj12 Rv | GCACCTTGCCATTGCCAAA |
| Kcnq1 Fw | GATCAGTCCATCGGGAAGCC |
| Kcnq1 Rv | GGTCCAGTTGTGTCACCTTGT |
| Slc9a1 Fw | AACGGCTGCGGTCCTATAAC |
| Slc9a1 Rv | CGAGACATGGTGGGTGAGTC |
| Scn1b Fw | AACACCAGCGTCGTCAAGAA |
| Scn1b Rv | TTCCGAGGCATTCTCTTGTGC |
| Scn2b Fw | CCTTGGTCCCTCAATCACCC |
| Scn2b Rv | ACTGTGACTTCCATGCTCCG |
| Scn3b Fw | CTTCCTCACCTTGTGGCTGT |
| Scn3b Rv | GCACTCAGATCACCTCAAGTCA |
| Scn5a Fw | TCTTCCGGTTCAGTGCCACC |
| Scn5a Rv | GGATGGTGCACATGATGAGCATG |
| U6 Fw | CTCGCTTCGGCACA |
| U6 Rv | AACGCTTCACGAATTTGCGT |
| hsa-miR-1-3p | TGGAATGTAAAGAAGTATGTAT |
| hsa-miR-133a-3p | TTTGGTCCCCTTCAACCAGCTG |
| hsa-miR-195-5p | TAGCAGCACAGAAATATTGGC |
| hsa-miR-24-3p | TGGCTCAGTTCAGCAGGAACAG |
| hsa-miR-26a-5p | TTCAAGTAATCCAGGATAGGCT |
| hsa-miR-26b-5p | TTCAAGTAATTCAGGATAGGT |
| hsa-miR-328-3p | CTGGCCCTCTCTGCCCTTCCGT |

**MIFlowCyt-EV of “MicroRNAs in Extracellular Vesicles released from Epicardial Adipose Tissue Promote Arrhythmogenic Conduction Slowing”**

**Flow cytometry**

**Experimental design**

The aim of flow cytometry (A60-Micro, Apogee Flow Systems, Hemel Hempstead, UK) experiments was to measure the concentrations of extracellular vesicles (EVs) released from epicardial and subcutaneous adipose tissue (EAT, SAT) in conditioned culture medium from adipose tissue samples collected from 7 patients to investigate whether EAT and SAT secretome (EATs, SATs) show different concentrations of EVs. We hypothesized that EAT secretes a larger number of EVs than SAT.

All samples were measured using an autosampler, which facilitates subsequent measurements of samples in a 96-well plate. For this study, samples were measured on two different days (03/01/2021 for 3 patient samples and 04/05/2022 for 4 patient samples). Each day a buffer-only control, antibody in buffer controls, and isotype controls corresponding to the labels in the well plate were measured. Flow rate and scatter calibrations were performed daily. Fluorescence calibration was performed twice during this time period. To automatically determine optimal sample dilutions, apply calibrations, determine and apply gates, generate reports with scatter plots and generate data summaries, we used custom-built software (MATLAB R2020b, Mathworks, Natick, MA, USA).

**Sample dilutions**

Samples containing submicrometer particles require different dilutions to (1) avoid swarm detection [1] and (2) achieve statistically significant counts within a clinically applicable measurement time. Although serial dilutions are recommended to find the optimal dilution, we consider serial dilutions unfeasible in clinical research studies. Therefore, we developed a procedure to estimate to optimal sample dilution (section 1.2 of <https://doi.org/10.6084/m9.figshare.c.4753676>). In sum, we showed that for our flow cytometer and settings used, a count rate ≤ 5.0∙10^3^ events second unlikely results in swarm detection.

Pre-staining control and SATs samples were undiluted, while EATs samples were undiluted, 1.5x diluted or 2x diluted, followed by an additional 11.125x dilution post-staining, resulting in count rates between 18 and 3844 events per second. More research is required, however, to confirm that the count rate can be used as a benchmark to avoid swarm detection for a given sample type.

**EV staining**

EVs were stained with antibodies. Prior to staining, antibodies were diluted in DPBS and centrifuged at 18,890 g for 5 min to remove aggregates. **Supplemental Table 5**  shows an overview of the used reagents and antibody concentrations during staining. Each sample was single labelled with CD9-PE, CD63-PE or Lactadherin-FITC. To stain, 20 μL of pre-staining diluted EATs and SATs samples was incubated with 2.5 μL of antibodies or isotype controls and kept in the dark for 2 h at room temperature. Post-staining, samples were diluted 11.125-fold in 200 μL of DPBS to decrease background fluorescence from unbound reagents.

**Buffer-only control**

Each measurement day at least 1 well with DPBS was measured with the same flow cytometer and acquisition settings as the samples. The mean count rate was 16 events per second, which is substantially lower than the count rates obtained for EATs/SATs samples (18 – 3,844 events per second).

**Buffer with reagents control**

Each 96-well-plate contained a buffer with reagent control for each reagent (**Supplemental Table 5**), which was measured with the same flow cytometer and acquisition settings as the samples. For all reagents, the mean count rate was between 14 - 22 events per second, which is in the same range as the buffer-only control.

**Unstained controls**

Unstained controls were measured with the same flow cytometer and acquisition settings as the stained samples, resulting in 15 – 3,427 events per second for EATs/SATs samples.

**Isotype controls**

**Supplemental Table 5** shows an overview of the used isotype controls, which were added to a selection of samples. For 21 control/EATs/SATs samples, we obtained an average of 5.5 x10^4^ IgG1-PE+ events with a diameter 160 nm - 1,000 nm per measurement. For comparison, the average concentration was 2.61x10^5^ CD63-PE+, 4.46x10^5^ CD9-PE+, 5.20x10^6^ Lactadherin-FITC+ events per mL with a diameter 160 nm - 1,000 nm were obtained in the experiments using EATs/SATs samples.

**Detergent-lysis controls**

20 μL of 10% NP-40 Detergent Solution were added to 2 of the EATs and 2 of the SATs labelled sample for detergents control analysis. Results are provided in **Supplemental Figure 6** and show that the detergent step removes EV signal in EATs and SATs.

**Trigger channel and threshold**

Based on the buffer-only control (38 events s^-1^), the acquisition software was set up to trigger at 14 or 24 arbitrary units (a.u.) SSC, which is equivalent to an SSC cross section of 8 or 10 nm^2^ (Rosetta Calibration, v1.29, Exometry, Amsterdam, The Netherlands), respectively. Both trigger thresholds enable detection of EVs with a diameter of 160 nm, which is the lower boundary of one of the applied gates.

**Flow rate quantification**

The A60-Micro is equipped with a syringe pump with volumetric control, which we checked on a daily basis using Apogee Mix and ApoCal Mix. Here, we assumed that the flow rate is equal to the adjusted flow rate of 3.01 µL/min for all measurements.

**Fluorescence calibration**

Calibration of the fluorescence detectors from arbitrary units (a.u.) to molecules of equivalent soluble fluorochrome (MESF) was accomplished using SPHERO Easy Calibration Fluorescent Particles (AK01, Spherotech Inc., Irma Lee Circle, IL, USA) and Quantum^TM^ MESF Kits (13734, Bangs Laboratories Inc., Fishers, IN, USA).

Calibration of the FITC and PE detector was performed once in the time period of these experiments. For each measurement, we added fluorescent intensities in MESF to the flow cytometry data files by custom-built software (MATLAB R2020b) using the following equation:

| $\text{I(MESF)}={10}^{a\cdot\log_{10} \text{I(a.u.)}+b}$ | Equation S1 |
| --- | --- |

where I is the fluorescence intensity, and *a* and *b* are the slope and the intercept of the linear fits, respectively, see **Supplemental Table 6** .

**Light scatter calibration**

We used Rosetta Calibration to relate scatter measured by FSC or SSC to the scattering cross section and diameter of EVs. Fig. S7 shows print screens of the scatter calibrations. We modelled EVs as core-shell particles with a core refractive index of 1.38, a shell refractive index of 1.48, and a shell thickness of 6 nm.

**MIFlowCyt checklist**

The MIFlowCyt checklist is added to **Supplemental Table 7**.

**EV number concentration**

The concentrations reported in the manuscript describe the number of particles (1) that exceeded the SSC threshold, corresponding to a side scattering cross section of 8 nm^2^ or 10 nm^2^, (2) that were collected during time intervals, for which the count rate did not a deviated more than 750 events/s of the median count rate, (3) with a diameter 160 nm – 1,000 nm as measured by SSC after light scatter calibration (section 1.11) and (4) are positive for PE or FITC, per mL of sample. The lower fluorescence gate is 148 MESF for CD63-PE, 130 MESF for CD9-PE, and 512 MESF for Lactadherin-FITC.

**Data sharing**

Data is available via: https://figshare.com/articles/dataset/Flow_cytometry_of_Epicardial_adipose_tissue_derived-extracellular_vesicles_/21695444

**
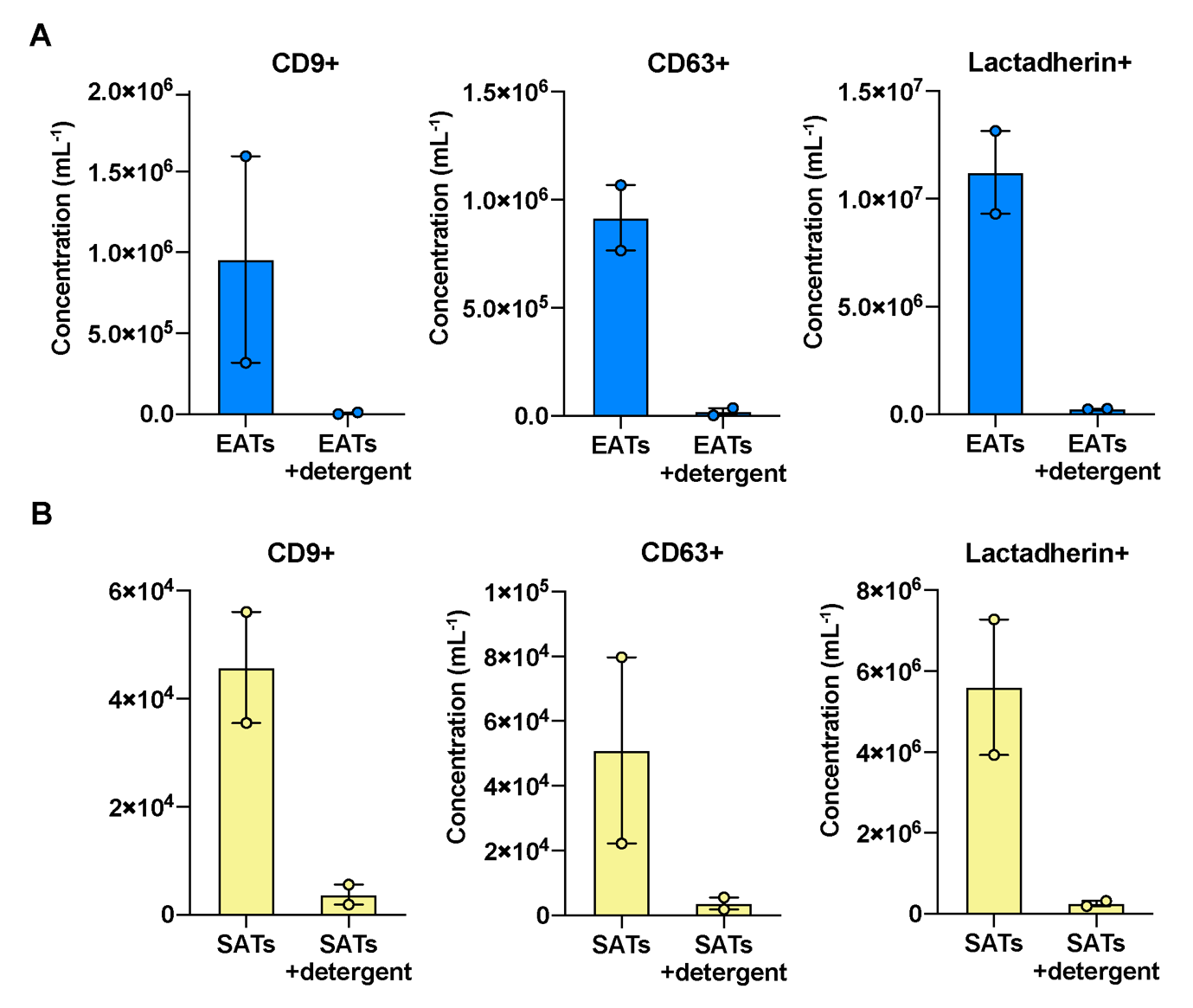
**

**Supplemental Figure 6. Detergent step removes EV signal in EATs and SATs.**

Concentrations of EVs per mL in EATs (A) and SATs (B) from n=2 patients, measured by flow cytometry after labelling EVs with anti-CD9-PE, anti-CD63-PE, and anti-Lactadherin-FITC, with and without detergent step (cf methods). Concentrations of EVs show the number of particles per mL of secretome (i) exceeding the side scattering threshold (ii) having a diameter <1,000 nm, and (iii) exceeding the fluorescent gate corresponding to the used labels. Data are mean ± standard error of the mean.


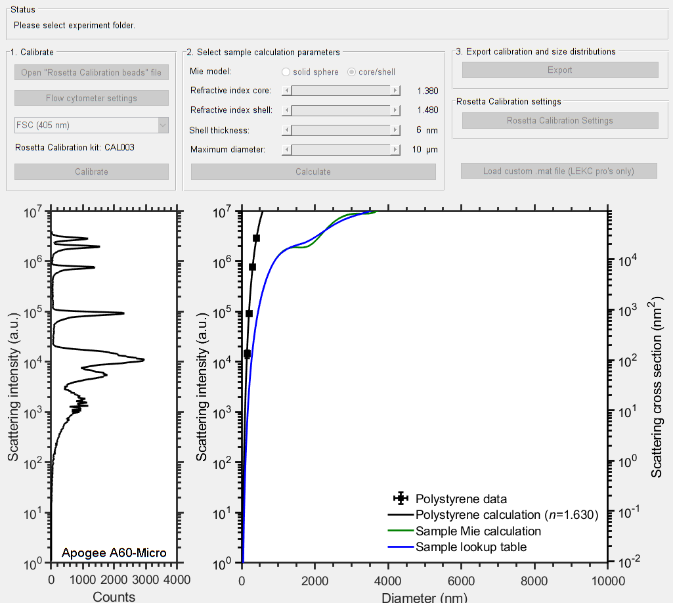

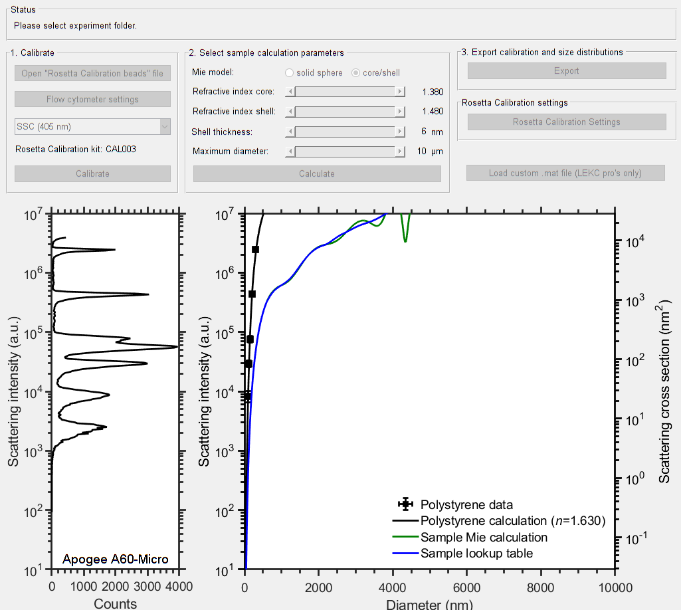


**Supplemental Figure 7. Scatter calibrations.** (A) Forward scatter and (B) side scatter calibration of the A60-Micro by Rosetta Calibration. To relate scatter to the diameter of EVs, we modelled EVs as core-shell particles with a core refractive index of 1.38, a shell refractive index of 1.48, and a shell thickness of 6 nm.

**Supplemental Table 5: Overview of staining reagents.**  Characteristics being measured, analyte, analyte detector, reporter, isotype, clone, concentration, manufacturer, catalog number and lot number of used staining reagents. The antibody concentration during measurements was 11.25-fold lower than the antibody concentration during staining.

| **Characteristic**  **measured** | **Analyte** | **Analyte detector** | **Reporter** | **Isotype** | **Clone** | **Concentration during staining (µg mL^-1^)** | **Manufacturer** | **Catalog number** | **Lot number** |
| --- | --- | --- | --- | --- | --- | --- | --- | --- | --- |
| Tetraspanin | Human  CD9 | Anti-human CD9 antibody | PE | IgG1 | M-L13 | 2.5 | BD bioscience | 555372 | 03905 |
| Tetraspanin | Human  CD63 | Anti-human CD62 antibody | PE | IgG1 | CLBGran/12 | 7.5 | Beckman Coulter | IM1914 | 28 |
| Phosphatidyl-L-serine | Bovine Lactadherin | Phosphatidyl-L-serine | FITC | IgG1 | n.a. | 10.4 | Prolytix | BLAC-FITC | MM0301 |
| Affinity for Fc receptor | Fc receptor | IgG1 | PE | n.a. | X40 | 7.5 | BD Bioscience | 345816 | 9309643 |

CD: cluster of differentiation; FITC: fluorescein isothiocyanate; IgG: immunoglobulin G; PE: phycoerythrin.

**Supplemental Table 6: Overview of fluorescence calibrations.**

|  | Calibration date | Slope | Intercept | R^2^ |
| --- | --- | --- | --- | --- |
| FITC | 2020-07-20 | 1.2291 | -1.4831 | 0.9988 |
| FITC | 2022-03-30 | 1.1823 | -2.1922 | 0.9993 |
| PE | 2020-07-20 | 1.0340 | -1.6142 | 0.9993 |
| PE | 2022-03-29 | 1.2538 | -2.7574 | 0.9916 |

FITC: fluorescein isothiocyanate; PE: phycoerythrin. R^2^: coefficient of determination.

**Supplemental Table 7 . MIFlowCyt checklist.**

| **Requirement** | **Please Include Requested Information** |
| --- | --- |
| 1.1. Purpose | To compare the EVs content between EATs and SATs obtained from 7 patients with atrial fibrillation |
| 1.2. Keywords | Epicardial adipose tissue, atrial fibrillation, extracellular vesicles, arrhythmias |
| 1.3. Experiment variables | EAT and SAT paired samples were collected from 7 different patients, on different dates. |
| 1.4. Organization name and address | Amsterdam University Medical Centers  Location Academic Medical Centre  Meibergdreef 9  1105 AZ Amsterdam  The Netherlands |
| 1.5. Primary contact name and email address | Auriane C. Ernault,  Amsterdam University Medical Centers  Location Academic Medical Centre  Meibergdreef 9  1105 AZ Amsterdam  The Netherlands  a.c.ernault@amsterdamumc.nl |
| 1.6. Date or time period of experiment | Measurement was performed on 03/01/2021 for 3 patients samples and 04/05/2022 for 4 patients samples |
| 1.7. Conclusions | EATs contains significantly more EVs than SATs |
| 1.8. Quality control measures | All samples were measured using an autosampler, which facilitates subsequent measurements of samples in a 96-well plate. Each well plate contained buffer-only controls (section S1.4), antibody in buffer controls (section S1.5), unstained controls (section S1.6) and isotype controls (section S1.7). The flow rate is calibrated with Apogee Calibration beads (Apogee Flow Systems, Hemel Hempstead, UK; section S1.9). Fluorescence detectors were calibrated (section S1.10) with SPHERO Easy Calibration Fluorescent Particles (AK01, Spherotech Inc., Irma Lee Circle, IL, USA) and Quantum^TM^ MESF Kits (13734, Bangs Laboratories Inc., Fishers, IN, USA). FSC and SSC were calibrated with Rosetta Calibration (v1.29, section S1.11). |
| 1.9 Other relevant experiment information | Samples were measured on 2 different days, in a time period of 17 months. |
| 2.1.1.1. Sample description | The samples analysed in this study are EATs and SATs. |
| 2.1.1.2. Biological sample source description | Adipose tissue was rinsed in phosphate-buffered saline, dissected to remove any blood or connective tissue, and minced into 1-2mm^3^ explants. Each explant piece was transferred to a well of a 96-well plate and incubated in 100 μL of culture medium for 24H at 37°C and 5% CO_2_ in order to stimulate adipokine and secretome release. Adipose tissue secretome was then collected.  For freeze-storage, secretome samples were transferred to 1.5 mL cryotubes and stored in -80˚C. Before staining, samples were thawed for 1 minute at 37°C. |
| 2.1.1.3. Biological sample source organism description | Human adipose tissue was collected as follow: seven patients with symptomatic, persistent or long-standing persistent atrial fibrillation (AF) undergoing video-assisted thoracoscopic pulmonary vein isolation (VATS-PVI) were recruited at the Amsterdam UMC, University of Amsterdam, between 2019 and 2022. Written informed consent was obtained from all participants. The study was conducted in accordance with the Declaration of Helsinki as revised in 2013 and with the Medical Research Involving Human Subjects Act (WMO) and other local laws and regulations. AF was documented on ECG, Holter or pacemaker electrogram at least once in the 12 months preceding surgery. Patients with left ventricular ejection fraction <30% and severe heart failure were excluded from the study. During VATS-PVI the left atrial appendage (LAA) was resected as standard procedure. The atrial tissue was collected and the EAT overlying the LAA was removed. A biopsy of approximately 1cm^3^ of subcutaneous adipose tissue (SAT) was obtained from one of the thoracoscopic entry sites. Adipose tissue was transported in phosphate-buffered saline to the laboratory. |
| 2.2 Sample characteristics | Epicardial and subcutaneous adipose tissue secretome are expected to contain EVs, lipoproteins and proteins. |
| 2.3. Sample treatment description | Samples were thawed and brought to the lab by manual transport in vertical position. Samples were prepared by double centrifugation and samples were stained. Please see section S1.3 for staining procedure. |
| 2.4. Fluorescence reagent(s) description | Please see **Supplemental Table 5**. |
| 3.1. Instrument manufacturer | Apogee, Hemel Hempstead, UK |
| 3.2. Instrument model | A60-Micro |
| 3.3. Instrument configuration and settings | Samples were analysed for 2 minutes at a flow rate of 3.01 μL/min on an A60-Micro, equipped with a 405 nm laser (100 mW), 488 nm laser (150 mW) and 638 nm laser (150 mW). The trigger threshold was set at SSC 14 or 24 a.u. corresponding to an SSC cross section of 8 nm^2^ or 10 nm^2^ (Rosetta Calibration), respectively. For FSC, the PMT voltages were 380 V or 470 V. For SSC, the PMT voltages were 350 V or 375 V. For all detectors, the peak height was analysed. FITC signals were collected with the 488-Green(Peak) detector (525/50 nm band pass filter, PMT voltage 520 V and 560 V). PE signals were collected with the 488-Orange(Peak) detector (575/30 nm band pass filter, PMT voltage 520 V and 450 V). |
| 4.1. List-mode data files | Data is available via FigShare.com:  10.6084/m9.figshare.21695444 |
| 4.2. Compensation description | No compensation was required because no fluorophore combinations were used that have overlapping emission spectra. |
| 4.3. Data transformation details | Fluorescence detectors were calibrated (section S1.10) with SPHERO Easy Calibration Fluorescent Particles (AK01, Spherotech Inc., Irma Lee Circle, IL, USA) and Quantum^TM^ MESF Kits (15036, Bangs Laboratories Inc., Fishers, IN, USA).  FSC and SSC were calibrated with Rosetta Calibration (v1.29, section S1.11). The concentrations reported in the manuscript describe the number of particles that fulfil the gating criteria per mL. |
| 4.4.1. Gate description | To automatically apply gates, generate pdf reports with scatter plots, and summarize the data in a table, custom-built software (MATLAB R2018b) was used. Please find below a description of the gates. First, events that were collected during seconds for which the count rate deviated less than 750 events/seconds from the median count rate were included. Second, events with a diameter 160 nm - 1,000 nm as measured by SSC after light scatter calibration (section S1.11) were included. Third, events positive for either FITC or PE were included. |
| 4.4.2. Gate statistics | The number of positive events was corrected for flow rate, measurement time and dilutions performed during sample preparation. |
| 4.4.3. Gate boundaries | The lower boundary of the diameter gate was 160 nm. The upper boundary of the diameter gate was 1,000 nm. The lower boundaries of the fluorescent gates were automatically determined (MATLAB R2020b). The lower fluorescence gate is 148 MESF for CD63-PE, 130 MESF for CD9-PE, and 512 MESF for Lactadherin-FITC. |

a.u.: arbitrary units; EVs: extracellular vesicles; FSC: forward scattering; SSC: side scattering.
